# Analysis of the electronic structure of the primary electron donor of photosystem I of *Spirodela* *oligorrhiza* by photochemically induced dynamic nuclear polarization (photo-CIDNP) solid-state nuclear magnetic resonance (NMR)

**DOI:** 10.5194/mr-1-261-2020

**Published:** 2020-11-13

**Authors:** Geertje J. Janssen, Patrick Eschenbach, Patrick Kurle, Bela E. Bode, Johannes Neugebauer, Huub J. M. de Groot, Jörg Matysik, Alia Alia

**Affiliations:** 1 Leiden Institute of Chemistry, Leiden University, 2300 RA Leiden, the Netherlands; 2 Organisch-Chemisches Institut, Universität Münster, 48149 Münster, Germany; 3 Institut für Analytische Chemie, Universität Leipzig, 04189 Leipzig, Germany; 4 EaStCHEM School of Chemistry, Biomedical Sciences Research Complex and Centre of Magnetic Resonance, KY16 9ST St Andrews, Scotland; 5 Institut für Medizinische Physik und Biophysik, Universität Leipzig, 04103 Leipzig, Germany; 6 Center for Multiscale Theory and Computation, Universität Münster, 48149 Münster, Germany

## Abstract

The electron donor in photosystem I (PSI), the chlorophyll dimer P700, is studied by photochemically induced dynamic nuclear polarization (photo-CIDNP) magic angle spinning (MAS) nuclear magnetic resonance (NMR) on selectively ${}^{13}C$ and uniformly ${}^{15}N$ labeled PSI core preparations (PSI-100) obtained from the aquatic plant duckweed (*Spirodela oligorrhiza*). Light-induced signals originate from the isotope-labeled nuclei of the cofactors involved in the spin-correlated radical pair forming upon light excitation. Signals are assigned to the two donor cofactors (Chl a and Chl a ') and the two acceptor cofactors (both Chl a ). Light-induced signals originating from both donor and acceptor cofactors demonstrate that electron transfer occurs through both branches of cofactors in the pseudo- $C_{2}$ symmetric reaction center (RC). The experimental results supported by quantum chemical calculations indicate that this functional symmetry occurs in PSI despite similarly sized chemical shift differences between the cofactors of PSI and the functionally asymmetric special pair donor of the bacterial RC of *Rhodobacter sphaeroides*. This contributes to converging evidence that local differences in time-averaged electronic ground-state properties, over the donor are of little importance for the functional symmetry breaking across photosynthetic RC species.

## 1 Introduction

In the process of oxygenic photosynthesis, electrons flow from photosystem II (PSII) to photosystem I (PSI); the nomenclature, however, follows the order of their discovery over time (Emerson and Chalmers, 1958; Govindjee and Rabinowitch, 1960). The X-ray structure of PSI from the prokaryotic system of cyanobacteria *Synechococcus (S.) elongatus* has been solved at 2.5 Å resolution as a trimeric supercomplex (Jordan et al., 2001). In the eukaryotic plant system of *Pisum sativum* (pea), the PSI structure has been resolved up to 3.4 Å resolution as a photosystem I and light-harvesting complex (LHCI; collectively PSI–LHCI; Ben-Shem et al., 2003). Cyanobacterial PSI contains 12 subunits with 96 chlorophyll (Chl) cofactors, while the plant complex consists of at least 17 subunits harboring over 170 Chls. In cyanobacteria, PSI is mostly observed as a trimer of monomeric PSI cores (Kruip et al., 1994; Fromme et al., 2001), while PSI in plants, red, and green algae is monomeric (Scheller et al., 2001; Kouril et al., 2005). Two functional moieties that can be distinguished in PSI are the photosystem I core that includes the redox active cofactors, and the peripheral light-harvesting complex (LHCI), which serves to increase the absorption cross section (Schmid et al., 1997; Amunts et al., 2009). While the structural organization of the redox centers is virtually identical in the structures obtained from *Pisum sativum* and *Synechococcus elongatus* (Jordan et al., 2001; Amunts et al., 2007), the LHCI complex shows a high degree of variability in size, subunit composition, and number or type of bound pigments. This variation allows each organism to adjust to its specific natural habitat (Croce et al., 2007; Wientjes et al., 2009). The PSI core complex prepared from plants is sometimes also denoted as the PS1-110 particle, referring roughly to the total number ( ∼110 ) of bound Chls (Mullet et al., 1980) and has a molecular weight of ∼300 kDa.

**Figure 1:**
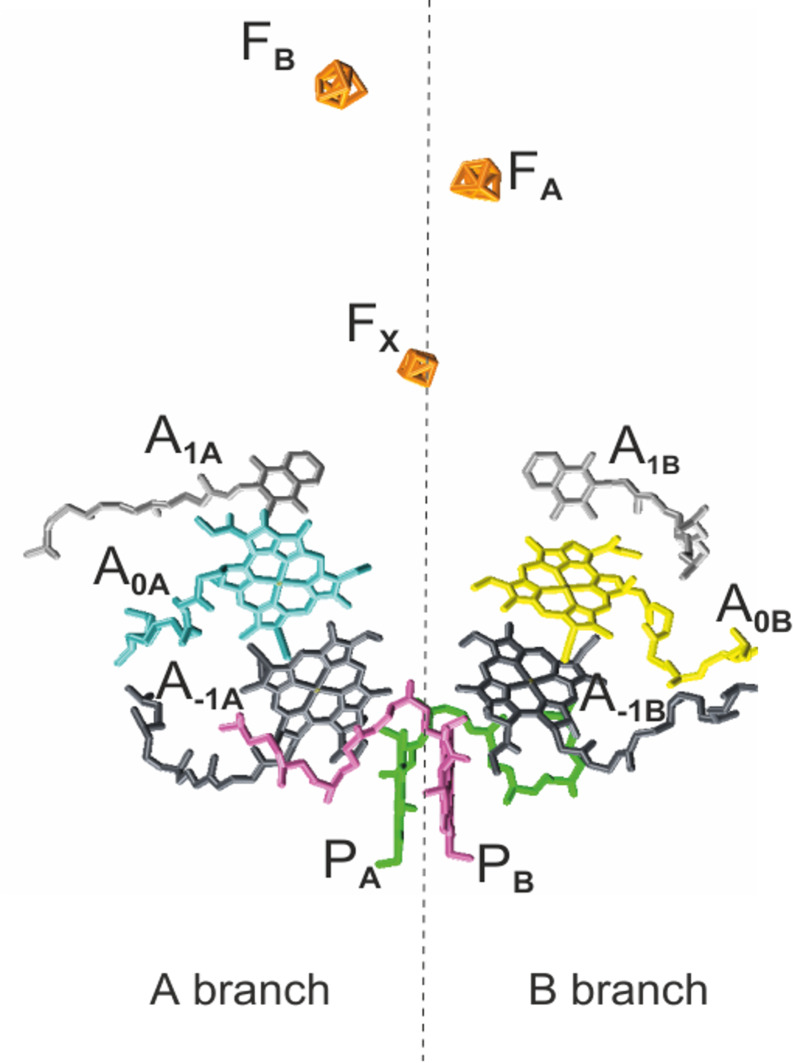
The arrangement of cofactors in the PS1 RC. Depicted in pink and green are the two central Chls, P ${}_{B}$ and P ${}_{A}$ , of the Chl a/ Chl a ' dimer. Furthermore, the RC contains two accessory Chl a (A ${}_{-1A}$ and A ${}_{-1B}$ ), two donor Chl a (A ${}_{0A}$ and A ${}_{0B}$ ), and two tightly associated phylloquinones (A ${}_{1A}$ and A ${}_{1B}$ ). Finally, there are three iron–sulfur [ 4Fe-3S ] clusters $({\text{F}}_{X},{\text{F}}_{A},$ and ${\text{F}}_{B})$ which function as terminal intrinsic electron acceptors. Both the A and B branches participate in electron transfer, with the relative activity depending mainly on the organism and the reduction conditions. PDB entry 2WSC (Amunts et al., 2009).

As in PSII and bacterial reaction centers (RCs), the cofactors in PSI are symmetrically arranged in two parallel chains relative to a pseudo- $C_{2}$ symmetry axis perpendicular to the membrane plane in which PSI is embedded in vivo (Fig. 1). Like type I bacterial RCs, PSI consists of six Chl cofactors, two quinones, and three iron–sulfur [4Fe-3S] clusters (F ${}_{X}$ , F ${}_{A}$ , F ${}_{B})$ acting as intrinsic electron acceptors. The F ${}_{A}$ and F ${}_{B}$ clusters operate in series and are bound to the PsaC subunit. The F ${}_{X}$ cluster is located at the interface between the PsaA and PsaB subunits, while the accessory Chls (A ${}_{-1A}$ and A ${}_{-1B})$ , the Chl acceptors (A ${}_{0A}$ and A ${}_{0B}$ ), and the quinone acceptors (A ${}_{1A}$ and A ${}_{1B}$ ) are bound to the PsaA (A branch) and PsaB (B branch). In comparison to their PSII quinone counterparts, A ${}_{1A}$ and A ${}_{1B}$ in PSI are more tightly associated with the protein backbone and are not as readily accessible for chemical-reducing agents (Srinivasan and Golbeck, 2009). With distances ranging between 15 and 40 Å, the cofactors in the PSI RC are more isolated from the surrounding antenna pigments than the cofactors in PSII and bacterial RC.

The electron donor, the heterodimeric P700, is, similarly to the F ${}_{X}$ cluster, located at the interface of both branches and consists of one chlorophyll a (Chl a ; P ${}_{B}$ ) and one Chl a ' (P ${}_{A}$ ), which is the C-13 ${}^{2}$ -epimer of Chl a . While P ${}_{A}$ forms hydrogen bonds to its protein environment, no hydrogen bonds are found on the P ${}_{B}$ side (Watanabe et al., 1985). The ratio of the spin-density distribution over the ${\text{P}}_{A}^{⚫\;+}/{\text{P}}_{B}^{⚫\;+}$ dimer exhibits significant diversity between species and conditions (Webber and Lubitz, 2001). Fourier transform infrared and electron paramagnetic resonance (EPR) spectroscopic studies on cyanobacterial PSI from *Synechocystis* indicated a ratio of electron spin density distribution in the range of 50:50 to 33:67 in favor of the P ${}_{B}$ (Breton et al., 1999). On the other hand, in spinach and *Thermosynechococcus (T.) elongatus*, ratios in the range of, respectively, 25:75 – 20:80 and 15:85 have been estimated (Davis et al., 1993; Käss et al., 2001). Electronic structure calculations suggested a ratio of 28:72 based on the coordinates taken from the high-resolution X-ray data of *T. elongatus* and indicate the hydrogen bonding of the P ${}_{A}$ Chl, the asymmetry in molecular geometry (Chl a / Chl a '), and minor differences in the protein environment as being the main factors influencing the relative spin density distribution over P ${}_{A}$ and P ${}_{B}$ (Saito et al., 2011).

Calculations making use of the frozen density embedding (FDE) technique on the primary electron donor of PSI in *S. elongatus*, including a large part of the protein environment, resulted in 76 % of the spin density being localized on P ${}_{B}$ (Artiukhin et al., 2020). Using FDE instead of the conventional Kohn–Sham density functional theory (DFT) can improve the description of the interaction of the electron donor with the protein matrix and the spin localization, as it avoids certain problems arising from the self-interaction error. The calculated spin populations were in good agreement with the references (Saito et al., 2011) and available experimental data obtained with ${}^{13}C$ photochemically induced dynamic nuclear polarization (photo-CIDNP) magic angle spinning (MAS) nuclear magnetic resonance (NMR; Alia et al., 2004).

While the donor in PSII is the strongest oxidizing agent known in living nature, P700 is optimized to provide a strong reducing force, which is required for the formation of nicotinamide adenine dinucleotide phosphate (NADPH). With a potential of approximately -1.2 V, P700 ${}^{⚫+}$ is probably the strongest reducing entity found in living systems (Ishikita et al., 2006).

Whereas in type II bacterial RCs and PSII electron transfer (ET) proceeds along only one of the two pseudosymmetric branches, over the past few years evidence has been accumulated to show that both branches are active in ET in PSI, resulting in a two-sided ET often denoted as bidirectional ET (see Santabarbara et al., 2010). Since femtosecond optical studies indicated the accessory Chl A ${}_{-1}$ to be the primary electron donor (Holzwarth et al., 2006; Müller et al., 2010), the structural asymmetry of the P700 Chl a / Chl a ' dimer is no longer a convincing argument against the participation of both branches in ET. A possible reason for the occurrence of the two-sided ET in type I systems as PSI and RCs of heliobacteria (Thamarath et al., 2012b) but not in type II systems as PSII and purple bacterial RC might be that the quinones in type II RCs function as a two-electron gate, with a mobile quinone on the inactive branch being used as a terminal acceptor (Müh et al., 2012). In type I systems, on the other hand, the iron–sulfur clusters act as terminal acceptors, while the quinone serves as an intermediary in electron transfer, making two-sided ET feasible. While consensus on the two-sided nature of ET in both prokaryotic and eukaryotic PSI has been reached (Fairclough et al., 2003; Redding et al., 2007), the molecular details controlling the ET pathways are not yet fully elucidated (Berthold et al., 2012). The relative activity of the two branches is in favor of the A branch but seems to vary among different organisms ranging from ∼3 to 2 in green algae (Holzwarth et al., 2006; Li et al., 2006) and ∼3 –4 to 1 in cyanobacteria (Ramesh et al., 2004; Dashdorj et al., 2005). The relation between the activity of the ET pathways and the electron (spin) density distribution between the two parts of the donor is not understood. In addition, the reducing conditions of the quinones appear to affect the relative branch activity with, e.g., ET in *Synechococcus lividus* occurring solely along the B branch at low temperatures (100 K) and strongly reducing conditions (Poluektov et al., 2005). Hence, the factors inducing the initial asymmetry are not yet understood.

**Scheme 1:**
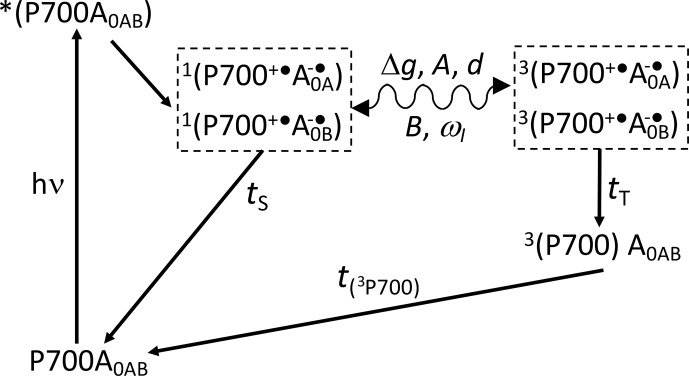
Reaction cycle in PSI, with reduced F ${}_{X}$ acceptor and electron transfer, over both branches of cofactors A and B. After the absorption of a photon, electron transfer occurs from the P700 donor to the primary acceptors A ${}_{0}$ . Upon chemical prereduction of F ${}_{X}$ , the electron transfer becomes cyclic. Due to spin conservation, the spin-correlated radical pair (SCRP) are formed in a pure singlet state. The SCRP in its singlet state can either recombine to the diamagnetic ground state or undergo coherent singlet–triplet interconversion to its electronic triplet state. This interconversion relies on the difference in the g values of the two electrons, Δg , and the hyperfine interaction with magnetic nuclei. According to the radical pair mechanism (RPM), this influence of the nuclei on the radical pair dynamics leads to spin sorting and, hence, to enrichment of nuclear spin states in the two decay channels. In frozen samples, three-spin mixing (TSM) produces nuclear hyperpolarization based on the secular part of the hyperfine interaction, A , the coupling between the two electrons, d , the pseudosecular hyperfine coupling, B , and the nuclear Zeeman frequency, $\omega_{I}$ . Furthermore, as caused by the different kinetics of the two decay channels ( $T_{S}$ vs. $T_{T})$ , the differential decay (DD) mechanism for nuclear spin hyperpolarization occurs. From the triplet state of the SCRP, a molecular donor triplet state is formed, which decays with a triplet lifetime, T ( ${}^{3}$ P700), of ∼3 µs (Polm and Brettel, 1998), making the occurrence of the differential relaxation (DR) mechanism unlikely.

To further investigate the functional symmetry breaking in PSI, we have studied isotope-labeled PSI-110 samples from duckweed with photo-CIDNP MAS NMR spectroscopy. Photo-CIDNP MAS NMR spectroscopy is an analytical method (for a review, see Matysik et al., 2009; Bode et al., 2013) informing on the molecules involved in spin-correlated radical pairs in both their electronic ground state (using NMR chemical shift information) and radical pair state (by photo-CIDNP intensities). The method is based on the solid-state photo-CIDNP effect, discovered in bacterial RCs in 1994 (Zysmilich and McDermott, 1994), occurring in spin-correlated radical pairs (SCRPs) in an immobile matrix upon cyclic ET. The NMR signal is enhanced by up to a factor of 80 000 (Thamarath et al., 2012a). The effect requires a cyclic reaction process that is introduced by the prereduction of the acceptor site. Scheme 1 shows such a reaction cycle for PSI. In the electronically excited state of the donor (P700 ${}^{*}$ ), an electron is transferred to the primary acceptor (A ${}_{0}$ ). The radical pair is formed in the singlet state and undergoes intersystem crossing to its triplet state. Magnetic coupling to nuclear spins alters the intersystem crossing rates for different nuclear spin states, leading to nuclear spin sorting on the singlet and triplet recombination pathways. Although both pathways return to the same product (i.e., the ground state P700-A ${}_{0}$ ), nuclear polarization is generated. The spin chemical mechanism has been probed by field-dependent (Thamarath et al., 2012a; Gräsing et al., 2017), time-resolved (Daviso et al., 2009a, 2010; Sai Sanker Gupta et al., 2014), and preparation-dependent (Matysik et al., 2000a; Daviso et al., 2011) experiments. Nuclear polarization arises from several different mechanisms operating in parallel. The classical radical pair mechanism (RPM; Kaptein and Oosterhoff, 1969; Closs and Closs, 1969) relies on spin sorting and produces transient nuclear polarization in both branches, canceling on the arrival of the population of the slower triplet decay channel. In addition, electron–nuclear spin dynamics in the radical pair state induce nuclear spin polarization through two solid-state mechanisms called three-spin mixing (TSM; Jeschke, 1997) and differential decay (DD; Polenova and McDermott, 1999), which remains for the period given by the $T_{1}$ relaxation time. Recently, Sosnovsky et al. (2016, 2019) reinterpreted these coherent solid-state mechanisms in terms of electron–electron–nuclear level crossings and level anticrossings. Furthermore, in the differential relaxation (DR) mechanism, also called cyclic reaction mechanism, the nuclear polarization of the triplet decay channel is quenched by the paramagnetic molecular triplet state, enhancing nuclear relaxation and making the cancellation of the RPM polarization incomplete (McDermott et al., 1998).

Various photosynthetic RCs of plants (Alia et al., 2004; Diller et al., 2005, 2007; Janssen et al., 2018), algae (Janssen et al., 2010, 2012), diatoms (Zill et al., 2017, 2019), purple bacteria (Prakash et al., 2007; Daviso et al., 2009b; Paul et al., 2019), heliobacteria (Thamarath et al., 2012b), green sulfur bacteria (Roy et al., 2008), and flavoproteins (Thamarath et al., 2010; Ding et al., 2019) have been analyzed with the photo-CIDNP MAS NMR method. Previously, the application of ${}^{13}C$ photo-CIDNP MAS NMR was restricted to the unlabeled and isolated PSI complex due to difficulties in obtaining selective ${}^{13}C$ labeling in plants. Based on the data obtained from natural abundance PSI, a first tentative assignment of the light-induced signals involved a single Chl a molecule, which is probably the P ${}_{B}$ cofactor of the donor P700 (Alia et al., 2004).

In this work, we report on the first selective incorporation of ${}^{13}C$ isotope labels in a PSI complex from duckweed (*Spirodela*). Backed by ${}^{15}N$ labeling and quantum chemical calculations, we have explored the photosynthetic machinery of PSI on ${}^{13}C$ and ${}^{15}N$ isotope-labeled preparations from duckweed by photo-CIDNP MAS NMR, aiming for the details of the electronic structure of the dimeric donor and the question of one- or two-sided ET. In addition to continuous illumination with white light, ${}^{13}C$ photo-CIDNP MAS NMR was induced by a 532 nm nanosecond flash laser.

## 2 Materials and methods

### 2.1 Photosystem I particle preparation

Duckweed plants were grown under aseptic conditions on half-strength Hunter's medium (Posner, 1967). For selective ${}^{13}C$ labeling of plants, 1.4 mM of δ -aminolevulinic acid, isotopically ${}^{13}C$ labeled at carbon position 4 (4-ALA; Cambridge Isotope Laboratories, Inc.), was added to the duckweed growth medium (half-strength Hunter's medium; pH 4.8). Plants were grown on labeled medium, under continuous light (20 $µE\;m^{-2}\;s^{-1}$ ), at 25 ${}^{\circ }$ C. The medium was continuously bubbled with sterile air containing 5 % ${\mathrm{CO}}_{2}$ . After 7 d, plants were harvested and used directly for sample preparation or frozen in liquid nitrogen and stored at -80 ${}^{\circ }$ C until use. The PSI complex containing ∼110 Chl / P700 (PS1-110 particles) was prepared according the method described by Alia et al. (2004).

### 2.2 Determination of the ${}^{15}N$ and ${}^{13}C$ label incorporation

Chl a were extracted from plants grown in ALA-supplemented half-strength Hunter's medium (labeled sample) and from unlabeled plants (reference sample), according to the procedure of Moran and Porath (1980). Plants were homogenized in MeOH. The methanolic solution was centrifuged for 5 min at 300×g . The green supernatant was separated and dried under a gentle stream of $N_{2}$ . The sample was resuspended in acetone, loaded on a cellulose column, and pure Chl a fractions were eluted with petroleum ether/acetone (7/3 v/v ). The solvent was evaporated under a $N_{2}$ flow, and the pure Chl a was stored at -20 ${}^{\circ }$ C in a dry nitrogen atmosphere. Label incorporation has been determined by mass spectrometry to be about 75 % for each particular carbon position of the 4-ALA isotope label pattern. For details, see the Supplement.

### 2.3 Photo-CIDNP MAS NMR experiments

The NMR experiments were performed by using DMX/AV-100, DMX/AV-200, DMX/AV-300 and DMX/AV-400 NMR spectrometers (Bruker GmbH, Karlsruhe, Germany). The samples were loaded into optically transparent 4 mm sapphire rotors. The PSI samples were reduced by the addition of an aqueous solution of a 10 mM sodium dithionite solution prepared in a 40 mM glycine buffer (pH 9.5) in an oxygen-free atmosphere. Immediately following the reduction, slow freezing of the sample was performed directly in the NMR probe inside the magnet under continuous illumination with white light. All spectra have been obtained at a sample temperature of 235 K and with a spinning frequency of 8 kHz.

The spectra were collected with a spin echo pulse sequence with a phase cycle of ( π/2 ) pulses under two-pulse phase modulation (TPPM) carbon–proton decoupling (Bennett et al., 1995). Photo-CIDNP MAS NMR spectra have been obtained using continuous illumination with a 1000 W Xenon arc lamp (Matysik et al., 2000b). The number of scans was 20 000, unless stated differently. The fitting of the collected spectra was performed using Igor Pro 6.01 (WaveMetrics, Inc., Portland, OR, USA). Based on the relative intensity of the signals, the electron spin density was calculated for the nitrogen assigned to the donor.

A pulsed nanosecond flash laser provides sufficient radiation intensity for time-resolved photo-CIDNP MAS NMR studies and does not decrease the time resolution that can be obtained in NMR experiments. The laser is operating with a repetition rate between 1 and 10 Hz. Using 1064 nm flashes of a Nd:YAG laser (Spectra-Physics Quanta-Ray INDI 40-10; Irvine, CA, USA) upon frequency doubling with a second harmonic generator (SHG), 532 nm laser flashes with pulse length of 6–8 ns and an energy between 20 and 150 mJ are produced.

### 2.4 Quantum chemical calculations

The structural models employed in our calculations were extracted from the crystal structure of PSI in plants (PDB entry 2WSC; Amunts et al., 2010), provided by the Protein Data Bank (Berman et al., 2000). Two different types of molecular models were considered, namely the so-called ISO models corresponding to the isolated cofactors extracted from the crystal structure. The binding pocket models, abbreviated as r32 and r34, were created by specifying radii of 3.2 and 3.4 Å around each atom of the cofactor of interest. All surrounding cofactors, water molecules, and amino acid residues with at least one atom within these radii were included explicitly into these models. For geometry optimizations, the DFTB3 (density-functional tight-binding, extended version) (Gaus et al., 2012) method within the AMS-DFTB (Amsterdam Modeling Suite) module from the ADF (Amsterdam Density Functional) 2019 package (Amsterdam; Velde et al., 2001) was used. The third-order parametrization for organic and biological system (3ob; Gaus et al., 2013; Kubillus et al., 2015) parameters from the corresponding Slater–Koster file were used. The optimizations were performed as a sequence of several steps that partly optimize the protein structure. For further details on the model setup and geometry optimization, see Sect. S2.1 and S2.2 in the Supplement. Graphical examples of the generated structures can be found in Sect. S5 in the Supplement.

NMR calculations of the binding pocket models of r32 or r34 were carried out within a subsystem DFT approach (Jacob and Visscher, 2006), using the TZP (triple-zeta polarized) (van Lenthe and Baerends, 2003) basis set and the Perdew–Wang (referred to as PW91 hereafter; Perdew and Wang, 1991; Perdew et al., 1992) XC (exchange—correlation) functional with the conjoint (Lee et al., 1991) kinetic energy functional Perdew–Wang kinetic (PW91k; Lembarki and Chermette, 1994). ${}^{15}N$ chemical shifts were calculated with respect to the ammonia shieldings, while ${}^{13}C$ chemical shifts were calculated with respect to tetramethylsilane (TMS; for further details, see Sect. S2.3 in the Supplement). Ring current effects of other subsystems were considered by calculating nuclear independent chemical shifts (NICSs), following Jacob and Visscher (2006). For further details on the NMR calculations, see Sect. 2.3–2.5.

## 3 Results and discussion

### 3.1 ${}^{15}N$ photo-CIDNP MAS NMR

**Figure 2:**
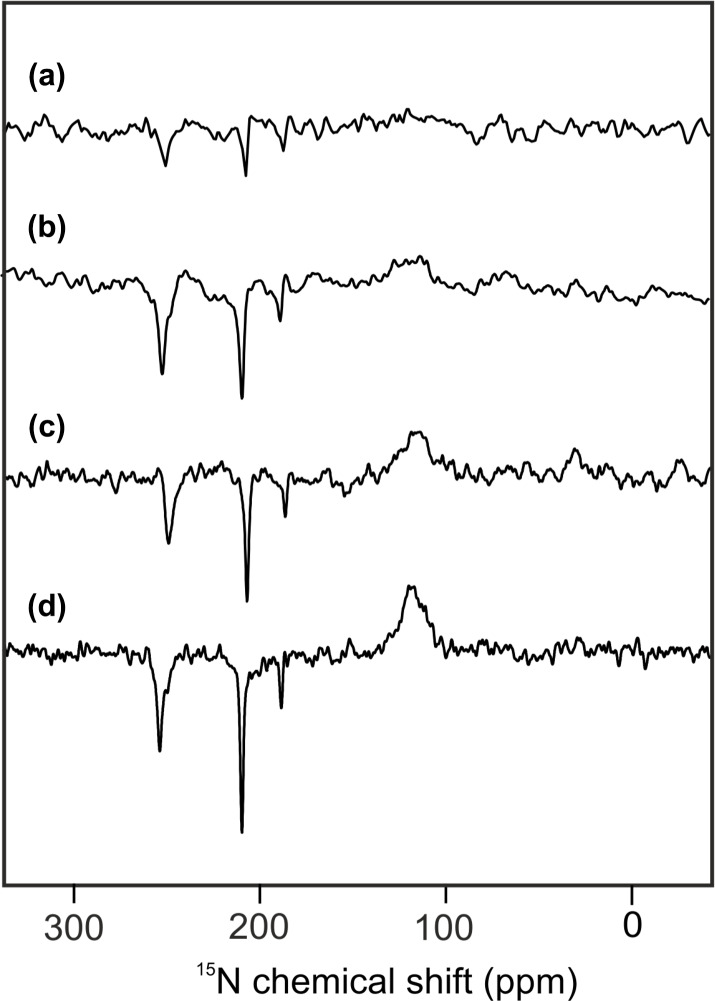
${}^{15}N$ photo-CIDNP MAS NMR spectra obtained from the same sample of uniformly ${}^{15}N$ labeled PSI-110 particles of duckweed measured at magnetic field strengths of **(a)** 2.35 T, **(b)** 4.7 T, **(c)** 7.1 T, and **(d)** 9.4 T. All spectra were obtained with a MAS frequency of 8 kHz, a temperature of 235 K, a cycle delay of 4 s, and an illuminance of 320 kLux provided by a white Xenon lamp. The number of scans was kept constant.

Figure 2 shows ${}^{15}N$ MAS NMR spectra of uniformly ${}^{15}N$ labeled PSI-110 particles of duckweed obtained under continuous illumination with white light at magnetic field strengths of (a) 2.35, (b) 4.7, (c) 7.1, and (d) 9.4 Tesla. At higher fields, the signal of the amide backbone nitrogen of the protein becomes clearly visible at about 125 parts per million (ppm) as a broad peak. In addition, sharp light-induced emissive (negative) signals were observed to originate from the Chl a and Chl a ' cofactors involved in formation of a SCRP. All light-induced signals are emissive at all magnetic fields investigated, and the absolute intensity increases with the magnetic field strength. Previous numerical simulations suggest that the matching conditions of the enhancement mechanisms are best met at 9.4 Tesla (i.e., 400 MHz ${}^{1}H$ frequency), leading to maximum signal enhancement (Roy et al., 2007). The three emissive ${}^{15}N$ signals appear at 254 (strong, with shoulder at 250), 210 (very strong, with weak shoulder at 207) and 188 ppm (medium). The signals are in good agreement with previous ${}^{15}N$ photo-CIDNP MAS NMR data of PSI from duckweed and spinach obtained at 4.7 T (Janssen et al., 2012) and can be conveniently assigned to a Chl a cofactor, with signals at 247.0, 189.4, 206.5 and 186.6 ppm in the solution of NMR for N-IV, N-III, N-II and N-I, respectively (Boxer et al., 1974). The strongest signal belongs to a single N-II, and the second strongest originates from the N-IV nitrogen. It is not clear whether the third signal occurring at 188 ppm originates from either N-I or N-III. Since shoulders and asymmetries occur, it appears that the signals originate from multiple cofactors. Emissive signals can arise from either donor or acceptor cofactors, and therefore, the sign cannot indicate the site of origin of signals. Since a chemical shift assignment would allow one to recognize whether the signals originate from donor or acceptor, and, if from the donor, whether from Chl a or Chl a ', we performed quantum chemical calculations to estimate the chemical shifts of the different cofactors.

The calculated ${}^{15}N$ chemical shifts of the two donor cofactors P ${}_{A}$ and P ${}_{B}$ , and the two acceptor cofactors A ${}_{0A}$ and A ${}_{0B}$ , are shown in Tables S2.1 and S2.2, respectively. The general chemical shift pattern is well reproduced by the calculations; however, assignment of resonances to specific cofactors is not possible. A possible source of the deviations from the calculated NMR shifts arises from the use of the static crystal structure rather than averaging over conformations accessible during the protein dynamics. This could be assessed by performing a short molecular dynamics (MDs) simulation and calculating NMR shifts for an ensemble of structures. Such a treatment could give an indication of thermal effects on the structure and, thus, implicitly on the NMR shifts. However, dynamic effects happening on longer timescales, which may be relevant for the NMR shifts as well, would not be covered in this way. The simulation of a structural ensemble is, however, beyond the scope of this work. Also, the inclusion of a sphere of protein environment of 3.2 and 3.4 Å (r32 and r34 in Table S2.1 and S2.2 and Fig. S3.1 to S3.4 in the Supplement) does not allow for a conclusive assignment. Although there is a significant environmental effect predicted, the strongest experimentally observed signal, N-II at 210 ppm, might be tentatively assigned either to P ${}_{A}$ or to A ${}_{0B}$ , with very similar values. The experimental signal at 254 ppm (N-IV) might be tentatively assigned to the donor cofactors with significantly larger chemical shifts than the acceptor signals. The two epimeric cofactors forming the donor cannot be distinguished on the basis of calculated chemical shifts. Hence, PSI, having four similar cofactors which might be involved into the formation of the SCRP, does not allow for straightforward ${}^{15}N$ chemical shift assignment.

### 3.2 ${}^{13}C$ photo-CIDNP MAS NMR

**Figure 3:**
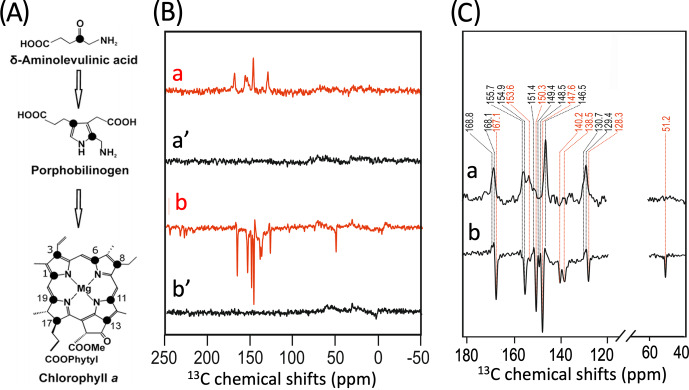
**(A)** Incorporation of $[4{-}^{13}C]-\mathrm{ALA}$ into cofactors (e.g., Chl a ) of PS1-110 of duckweed. The black dots indicate the positions of ${}^{13}C$ isotopes. **(B)** ${}^{13}C$ photo-CIDNP MAS NMR spectra obtained under continuous illumination (red) of 4-ALA labeled PS1-110 particles of duckweed at a magnetic field strength of 4.7 T **(a)** and 9.4 T **(b)**. Spectra **(a', b')** depicted in black originate from the corresponding experiments obtained in the dark. **(C)** Enlarged view of ${}^{13}C$ photo-CIDNP MAS NMR spectra of 4-ALA-labeled PS1-110 particles of duckweed obtained at 4.7 T **(a)** and 9.4 T **(b)**. Assigned signals are shown by the dashed lines, and emissive signals are in red.

To further characterize the individual cofactors of PSI involved in SCRP, we next performed ${}^{13}C$ photo-CIDNP MAS NMR on selectively ${}^{13}C$ labeled PSI. Previously, ${}^{13}C$ photo-CIDNP MAS NMR studies on plant PSI have been restricted to experiments on unlabeled preparations due to the difficulty of incorporating selective ${}^{13}C$ isotope labels into plant RCs. In the present study, we succeeded in selectively incorporating 4-ALA in PSI from duckweed with an isotope enrichment of 75 % for each particular carbon position of the 4-ALA isotope label pattern (Fig. 3A).

The ${}^{13}C$ NMR spectra in Fig. 3B are obtained from 4-ALA-labeled PSI-110 preparations at a magnetic field strength of 4.7 T ( ${}^{1}H$ frequency of 200 MHz; spectra a) and 9.4 T (400 MHz; spectra b) obtained under continuous light or in the dark. The spectra under illumination show several light-induced signals (shown in red) which are not observable in the dark (shown in black). The light-induced signature, however, is very different at the two magnetic fields. While the light-induced signals obtained at 4.7 T are entirely enhanced absorptive, at 9.4 T most of the signals appear emissive. Significant magnetic field effects have been observed for RCs of heliobacteria (Thamarath et al., 2012b) and purple bacteria (Thamarath et al., 2012a), and a similar dramatic sign change has been observed very recently in ${}^{13}C$ MAS NMR spectra of natural abundance PSII preparations from the diatom *Phaeodactylum tricornutum* (Zill et al., 2019). For unlabeled PSI-110 preparations of spinach (Alia et al., 2004), an entirely emissive envelope has been observed at 400 MHz. In the present study, however, some signals appear to turn positive, suggesting that ${}^{13}C$ isotope labeling indeed has some influence on the spin dynamics. The entirely emissive envelope observed at higher fields suggests the absence of contributions by the DR mechanism, which is reasonable due to the presence of carotenoids, implying that the solid-state photo-CIDNP effect relies on DD and TSM mechanisms. Since the ratio between TSM and DD is field dependent (Jeschke and Matysik, 2003), our data suggest that the TSM, expected to cause emissive signals as found for samples at natural abundance (Prakash et al., 2005), contributes more strongly, while the DD decays at higher fields.

A more detailed view of the light-induced signals is provided in Fig. 3C. The chemical shifts of the observed lines in the spectra are listed in Table 1. Careful examination of the spectra show that the emissive signals observed at a higher field (spectrum 3B in red) are canceled at a lower field, while the positive signals are visible in both spectra (in black). Therefore, it appears that the signals belong to two different sets. One might assume that one set originates from the donor and the other from the acceptor cofactors of PSI. Since the solid-state photo-CIDNP mechanisms DD and TSM require hyperfine anisotropy and occur on aromatic carbons, the occurrence of the emissive signal at 51.2 ppm at 9.4 T, originating from a C-17, the only aliphatic labeled position in the label pattern, is due to spin diffusion, i.e., polarization transfer from nearby ${}^{13}C$ -labeled aromatic carbons. The cancellation of this signal at 4.7 T might imply that at that field the nearby aromatic carbons also do not obtain enhancement.

**Table 1:** ${}^{13}C$ chemical shifts of the photo-CIDNP signals obtained at 4.7 and 9.4 Tesla in comparison to literature data. Assignments obtained from 4-ALA-labeled samples are given in italics. See the footnotes below the table for more details. In the reference work, carbons C-9 and C-11 could not be separated.

Carbon number	${}^{13}C$ chemical shifts				
*(4-ALA label)*	(ppm)				
	Chl a	Assignments			
		N.a. plant	4-ALA cyanobacteria	4-ALA plant	
			4.7 T	4.7 T	9.4 T
	δ ${}^{a}$	δ ${}^{b}$	δ ${}^{c}$	δ ${}^{d}$	δ ${}^{d}$
*19*	170.0	167.1 E	*166.9 E*	*168.5 A*	*168.8 A*
					*168.1 A*
					*167.1 E*
14	162.0	160.4 E			
*1*	155.9	158.4 E	*154.8 E*	≈ *155 A*	*154.9 E*
*6*	154.4			*153.6 A*	*153.6 E*
16	154.0	152.6 E			
4	150.7	149.9 E			
9	147.2	147.2 E			
*11*	147.2	147.2 E	*149.8 E*	*150.3 A*	*151.4 A*
			*147.6 E*		*150.3 E*
					*149.4 E*
					*147.6 E*
*8*	146.2	144.2 E	*144.2 E*	*146.5 A*	*146.5 A*
*3*	138.0	138.6 E	*138.6 E*		*140.2 E*
					*138.5 E*
2	136.1	≈136 E			
12	134.0				
7	133.4	≈132 E			
*13*	126.2			*130.7 A*	*130.7 A*
				*129.4 A*	*129.4 A*
					*128.3 E*
10	108.2	105.4 E			
15	102.8	105.4 E			
5	98.1				
20	93.3				
*17*	51.4		*53.9 E*		*51.2 E*

${}^{a}$ Boender et al. (1995) – data experimentally obtained from solid aggregates of Chl a . ${}^{b}$ Alia et al. (2004) – data experimentally obtained from isolated PSI particles from spinach. ${}^{c}$ Janssen et al. (2010) – data experimentally obtained from 4-ALA labeled whole cells of *Synechocystis* containing both PSI and PSII. ${}^{d}$ This work. Data experimentally obtained from 4-ALA labeled isolated PSI from duckweed. A – absorptive (positive); E – emissive (negative) signal intensity; N.a. – natural abundant isotope distribution; ppm – parts per million; numbers in italics – ${}^{13}C$ isotopically labeled in the 4-ALA pattern (Fig. 3).

All light-induced signals can be assigned conveniently to respective ${}^{13}C$ -labeled carbon positions of cofactors (indicated in italics in Table 1). Since there is no light-induced signal which requires assignment to a nonlabeled position, we assume that all observed signals originate from ${}^{13}C$ -labeled carbons. This assumption is reasonable considering the enrichment factor of 75 %. For several of the labeled ${}^{13}C$ positions, multiple signals are observed which support the conclusion from the ${}^{15}N$ data that several cofactors are observed. Three signals can be assigned to the carbons C-19 and C-13. For position C-11, four signals can be resolved. This observation strongly suggests that all four cofactors experience signal enhancement, implying that all four cofactors are involved in the spin-correlated radical pair and confirming that both electron transfer pathways are active. The alternating sign of the signals is typical for the magnetic field strength close to a turning point (see above).

To explore whether the assignment can be improved by attribution to individual cofactors occurring from the aromatic ${}^{13}C$ carbons, quantum chemical calculations have been performed for the bare cofactors and have included surrounding amino acids up to a shell of 3.4 Å (Table S2.3 and S2.4). For C-11, four experimental values of 151.4, 150.3, 149.4, and 147.6 ppm have been observed. The calculated shifts for C-11 span a similar range of about 5 ppm. In general, the calculated values for the other carbon positions confirm this finding. The differences between the four cofactors are in the range of about less than 5 ppm in PSI. The differences in chemical shifts between the two BChl cofactors of the special pair in *R. sphaeroides* were found to be slightly larger in previous studies (Schulten et al., 2002; Daviso et al., 2009b; Sai Sankar Gupta et al., 2014). However, this relatively limited difference in chemical-shift asymmetry cannot explain the fundamentally different functional asymmetry in the bacterial RC. Since the chemical shift refers essentially to time-averaged electronic ground state properties, it is tempting to conclude that the different behavior of donor dimers is encoded in the dynamic structure. This is corroborated by studies of the functional symmetry breaking involving the special pair in bacterial RCs, which is thought to originate from specific long-living cooperative modes for the semiclassical coherent mixing of the charge transfer character into the electronically excited state from which the electron is transferred (Thamarath et al., 2012a) and local differences in molecular dynamics affecting the electron–phonon coupling (e.g., Novoderezhkin et al., 2004; Wawrzyniak et al., 2011).

**Figure 4:**
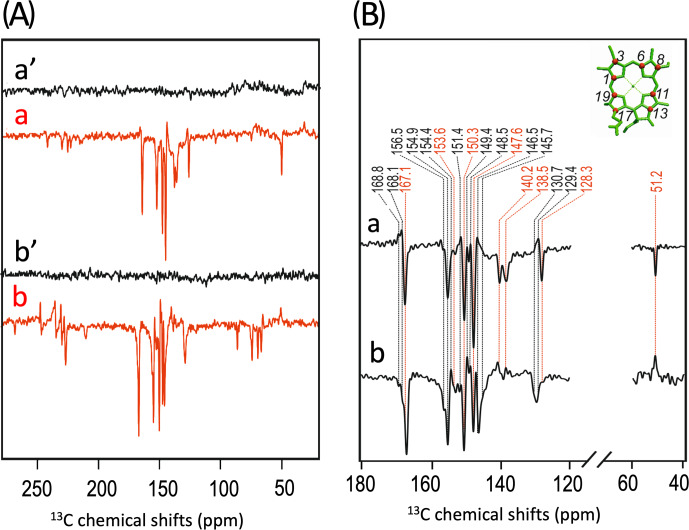
**(A)** ${}^{13}C$ photo-CIDNP MAS NMR spectra of 4-ALA-labeled PS1-110 particles of duckweed obtained at 9.4 T under continuous illumination **(a)** and under nanosecond laser flashes with zero delay **(b)**. Spectra **(a', b')** depicted in black originate from the corresponding experiments obtained in the dark. **(B)** Enlarged region of ${}^{13}C$ photo-CIDNP MAS NMR spectra of 4-ALA-labeled PS1-110 particles of duckweed obtained at 9.4 T under continuous illumination **(a)** and under nanosecond laser flashes with zero delay **(b)**. Assigned signals are shown by the dashed lines, and emissive signals are in red.

Figure 4 compares the ${}^{13}C$ photo-CIDNP MAS NMR spectrum induced by white light upon continuous illumination (spectrum 4Aa) with that induced by a 532 nm nanosecond flash laser (spectrum 4Ab). The magnified view of both light-induced spectra is shown in Fig. 4B. Remarkably, in the spectrum obtained by the laser flash experiment, several signals changed their sign. Enhanced absorptive signals turned emissive for the peaks at 168.8, 168.1 (both are assigned to C-19) and 129.4 ppm (C-13). On the other hand, several emissive signals become enhanced absorptive at 140.2, 138.5 (both arise from a C-3), and at 51.2 ppm (C-17). The different intensity patterns are due to differences in the enhancement mechanisms. Under steady-state illumination, the solid-state mechanisms TSM and DD produce the hyperpolarization which rely on anisotropic hyperfine interactions. In time-resolved experiments, the first detectable signals mainly refer to the singlet branch of the RPM and are based on isotropic hyperfine interactions. Equilibration of the polarization between the labeled carbons by spin diffusion occurs on a slower timescale (Daviso et al., 2009a).

## 4 Conclusions

There is experimental evidence that both the cofactors of the donor (P ${}_{A}$ and P ${}_{B})$ and both the potential acceptor cofactors (A ${}_{0A}$ and A ${}_{0B})$ carry the electron spin density of the spin-correlated radical pair. This confirms that both electron pathways in the PSI of duckweed are active and that the electron transfer does not occur exclusively in one branch. In addition, the time-averaged ground state electron density, as measured by the chemical shift, varies to a similar extent as in the functionally asymmetric special pair of RCs of *R. sphaeroides*. Our study suggests that the breaking of functional symmetry is not primarily due to local variation in time-averaged electronic ground-state properties at the donor site but, for instance, local and global electronic excited-state properties in conjunction with molecular dynamics.

## Supplement

10.5194/mr-1-261-2020-supplement The Supplement contains the following information: determination of the isotope incorporation, computational details, chemical shifts calculated by quantum chemical methods, the effect of the protein environment on the calculated NMR Shifts, and graphical examples of structures. The supplement related to this article is available online at: https://doi.org/10.5194/mr-1-261-2020-supplement.

## Data Availability

Original NMR data were obtained before 2013 and cannot be provided.
