# Supplementary material for: Analysis of the electronic structure of the primary electron donor of photosystem I of Spirodela oligorrhiza by photochemically induced dynamic nuclear polarization (photo-CIDNP) solid-state nuclear magnetic resonance (NMR)
Source: Magn Reson (Gott). 2020 Nov 13;1(2):261–74. doi: 10.5194/mr-1-261-2020 (PMC10655075; doi:10.5194/mr-1-261-2020)
Supplement: The Supplement contains the following information: determination of the isotope incorporation, computational details, chemical shifts calculated by quantum chemical methods, the effect of the protein environment on the calculated NMR Shifts, and graphical examples of structures. The supplement relat [file mr-1-261-supplement.pdf]

Supplement of Magn. Reson., 1, 261–274, 2020
https://doi.org/10.5194/mr-1-261-2020-supplement
© Author(s) 2020. This work is distributed under
the Creative Commons Attribution 4.0 License.

Open Access
MAGNETIC
RESONANCE

*Supplement of*

# Analysis of the electronic structure of the primary electron donor of photosystem I of *Spirodela oligorrhiza* by photochemically induced dynamic nuclear polarization (photo-CIDNP) solid-state nuclear magnetic resonance (NMR)

**Geertje J. Janssen et al.**

*Correspondence to:* Alia (a.alia@chem.leidenuniv.nl) and Jörg Matysik (joerg.matysik@uni-leipzig.de)

The copyright of individual parts of the supplement might differ from the CC BY 4.0 License.

## *1. Determination of the level of isotope labeling in Synechocystis sp. PCC 6803 and duckweed by LC-MS.*

Table S1.1. LCMS peak intensities and calculated $^{L}I_t$ and $P_n$ values for unlabeled and labeled Chl *a* isolated from *Synechocystis* sp. PCC 6803

| m/z | $^{U}I$ | $^{U}I_{\%}$ | $^{L}I$ | $^{L}I_t$ | $P_n$ |
|---|---|---|---|---|---|
| 893.5 | 429304.0 | 1 | 190318.8 | 190318.8 | 0.545 |
| 894.5 | 288949.2 | 0.67 | 245637.4 | 117540.6 | 0.336 |
| 895.5 | 73242.9 | 0.17 | 147672.0 | 36089.6 | 0.103 |
| 896.5 | 26567.0 | 0.06 | 56997.8 | 876.1 | 0.003 |
| 897.5 | 7339.1 | 0.02 | 16776.9 | 2756.2 | 0.008 |
| 898.5 | 1579.5 | 0.00 | 5400.6 | 1162.7 | 0.003 |
| 899.5 | 0.0 | 0.00 | 4324.3 | 782.6 | 0.002 |
| 900.5 | 0.0 | 0.00 | 0.0 | 0.0 | 0.000 |
| 901.5 | 0.0 | 0.00 | 0.0 | 0.0 | 0.000 |

Table S1.2. LCMS peak intensities and calculated $^{L}I_t$ and $P_n$ values for unlabeled and labeled Chl *a* isolated from duckweed

| m/z | $^{U}I$ | $^{U}I_{\%}$ | $^{L}I$ | $^{L}I_t$ | $P_n$ |
|---|---|---|---|---|---|
| 893.5 | 161481.0 | 1 | 118245.9 | 118245.9 | 0.289 |
| 894.5 | 120301.9 | 0.74 | 193086.8 | 104994.7 | 0.257 |
| 895.5 | 68342.5 | 0.42 | 173475.7 | 45211.2 | 0.111 |
| 896.5 | 9208.8 | 0.17 | 121856.1 | 36994.8 | 0.090 |
| 897.5 | 3323.8 | 0.02 | 75276.7 | 22594.0 | 0.055 |
| 898.5 | 1277.0 | 0.00 | 41455.4 | 6387.8 | 0.016 |
| 899.5 | 633.7 | 0.00 | 26502.6 | 10071.8 | 0.025 |
| 900.5 | 0.0 | 0.00 | 28208.2 | 16712.9 | 0.041 |
| 901.5 | 0.0 | 0.00 | 64674.5 | 47596.7 | 0.116 |

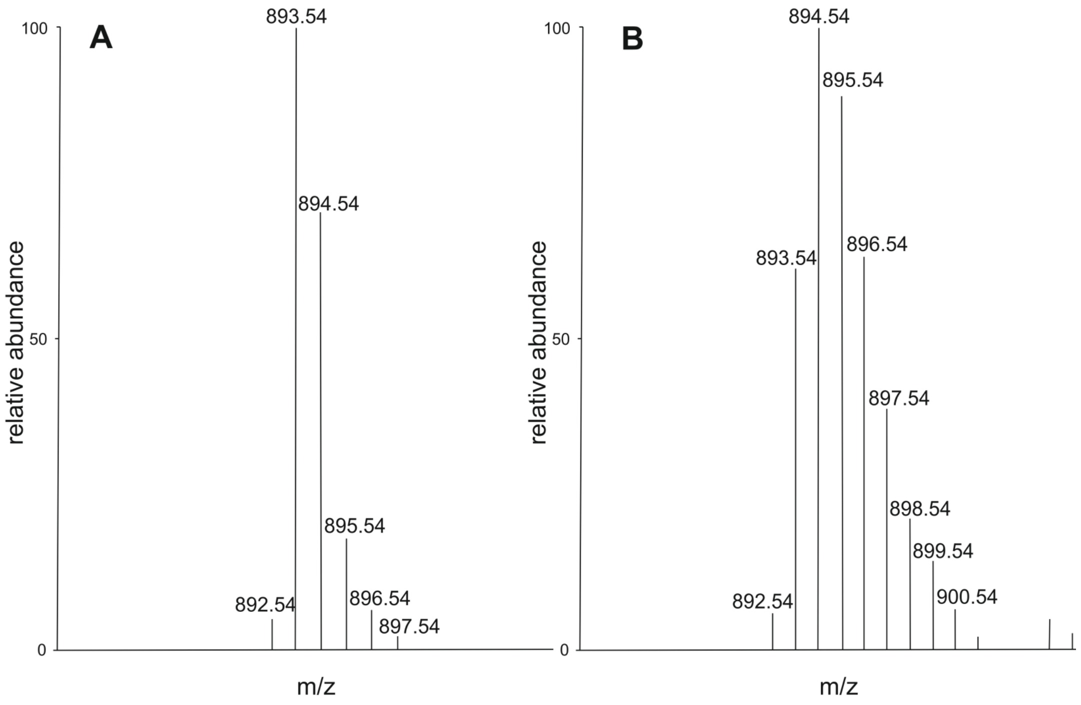


**Figure S1.1:** $^{13}C$ isotope incorporation determined by LCMS for Chl *a* isolated from duckweed leaves grown on unlabeled substrate (A) and with the $^{13}C$ 4-ALA precursor in the medium (B).

# 2 Computational Details

## 2.1 Model Setup

The desired models were extracted from the crystal structure of Photosystem I in plants (PDB entry 2WSC (Amunts et al., 2010), provided by the Protein Data Bank (Berman et al., 2000). The protein and non-protein part of the models was processed separately. The *Reduce* program (Word et al., 1999) was used to add missing hydrogen atoms of co-factors. This procedure was not accurate enough to add hydrogen atoms of water molecules, why they were added manually with *Avogadro* (Hanwell et al., 2012). Missing hydrogens of the protein were added using *CHARMM22* topology files (MacKerell et al., 1998; MacKerell et al., 2004) with the *Automatic PSF Builder* within the *VMD* (Humphrey et al., 1996) program. Additionally, cut protein bonds were saturated with neutral groups -C(O)$CH_3$ and -$NH_2$ as N- and C-terminus, respectively. The values of bond distances for the C- and N- termini were manually adjusted. The distances of corresponding groups in the crystal structure were added to a modified topology file (see Fig. S2.1). Binding pocket models of PSI were created by specifying radii of 3.2 and 3.4 Å around each atom of the co-factor of interest. All surrounding co-factors, water molecules, and amino acid residues with at least one atom within these radii were included explicitly into the models. Addition of missing hydrogens and cut bond saturation were conducted in a similar fashion as for smaller models considered in this work (see above). The binding pocket models are indicated by abbreviations corresponding to the chosen radii "r32" and "r34", while the isolated co-factor models are abbreviated "iso".

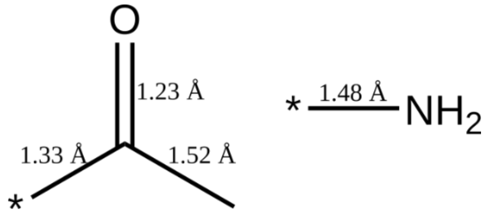


**Figure S2.1: Bond distances for the N- and C-termini.**

## 2.2 Geometry setup

To assess the quality of the calculated chemical shifts, the structure of $P_A$ was extracted from the crystal structure, truncated and processed according to Sec. 2.1. The molecular structure was then fully optimized using the *Turbomole v7.4.1* (Ahlrichs et al., 1989; TURBOMOLE, 2019) program package. This model will be called "isoOpt" in the following. For the optimization the def2-SVP basis set (Schäfer et al., 1992) and PBE0 functional (Perdew et al., 1996a; Adamo and Barone, 1999) were used. Additionally, the resolution-of-the-identity approximation in conjunction with the auxiliary Coulomb fitting basis (Schäfer et al., 1992; Schäfer et al., 1994) was enabled. Dispersion corrections were taken into account with the D3 correction with Becke–Johnson damping (Grimme et al., 2010; Grimme et al., 2011). Secondly, the structure of $P_A$ was extracted from the crystal structure and optimized using the DFTB3 (Gaus et al., 2012) method, which is described in detail in the following. This model will be called "Pa extract".

For all other geometry optimizations, the DFTB3 (Gaus et al., 2012) method within the *AMS-DFTB* module from the *ADF* 2019 package (Amsterdam; Velde et al., 2001) was used. The "Third-Order Parametrization for Organic and Biological Systems" (3ob) (Gaus et al., 2013; Kubillus et al., 2015) parameters from the corresponding Slater–Koster file were used. The optimization was carried out within two steps, where in the first step the coordinates corresponding to hydrogen atoms were optimized, while all other nuclear coordinates were kept fix. In the second step for each Chl co-factor the atoms C-3$^1$, C-3$^2$ and their attached hydrogen atoms were optimized while all other coordinates were kept fix. This step was done, to encounter the poor quality of this type of double bond in the crystal structure, where atoms C-3, C-3$^1$ and C-3$^2$ are oriented linearly. The general two-step procedure ensured to keep the relative arrangement of co-factors and environment residues, while the co-factor's geometry is partly relaxed in the presence of the protein pocket.

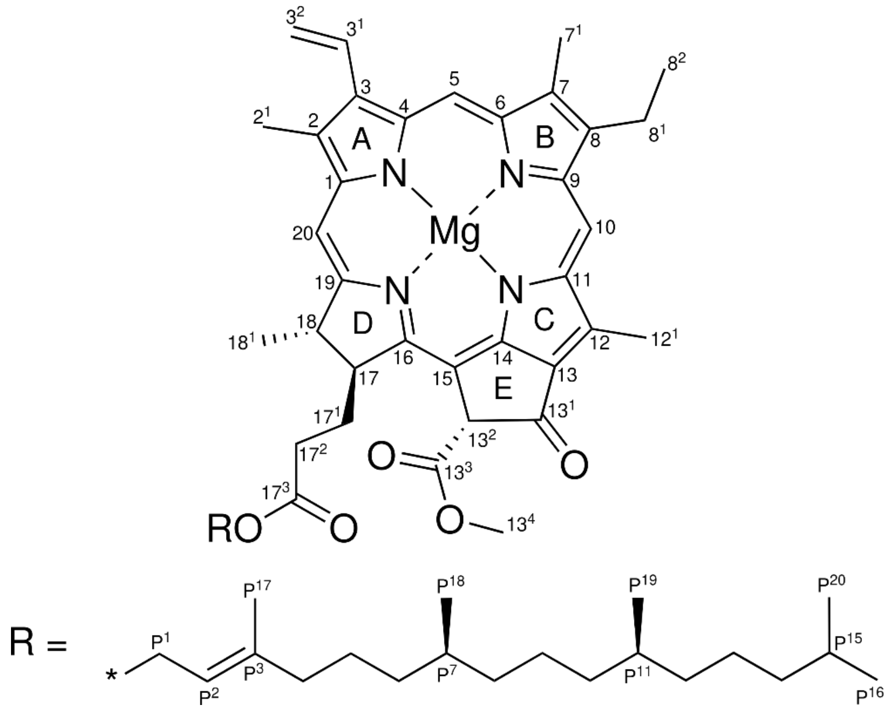

**Figure S2.2: Lewis structure of Chl *a*. The atomic numbering is given according to IUPAC. Nitrogens N-I, N-II, N-II and N-IV are localized in pyrrole rings A, B, C and D, respectively.**

## 2.3 NMR Calculations

For the "iso" models $^{15}N$ and $^{13}C$ nuclear magnetic shieldings were calculated using the KT2 (Keal and Tozer, 2003) exchange-correlation functional and a TZP (Perdew et al., 1992) basis set from the *ADF* 2019 package (Amsterdam; Velde et al., 2001) library. The numerical quality of the density fit and grid construction procedures were set to "good". For binding pocket models, labeled as "r32" or "r34", calculations were carried out within a subsystem DFT approach (Jacob and Visscher, 2006; Jacob and Neugebauer, 2014; Wesolowski et al., 2015) using the TZP (van Lenthe and Baerends, 2003) basis set and the PW91 (Perdew

and Wang, 1991; Perdew et al., 1992) exchange-correlation functional with the conjoint (Lee et al., 1991) kinetic-energy functional PW91k (Lembarki and Chermette, 1994). Mutual relaxations of subsystem densities were accounted for using 3 freeze-and-thaw (FaT) cycles (Wesolowski and Weber, 1996). $^{15}N$ chemical shifts were calculated with respect to the ammonia shieldings, while $^{13}C$ chemical shifts were calculated with respect to tetramethylsilane (TMS). Therefore, both molecules were optimized using the KT2 (Keal and Tozer, 2003) exchange-correlation functional and a TZP (Perdew et al., 1992) basis set from the *ADF* 2019 package (Amsterdam; Velde et al., 2001) library. $^{15}N$ as well as $^{13}C$ nuclear magnetic shieldings were calculated, where the chemical shift was calculated by $\delta_i = \sigma_{ref} - \sigma_i$, where $\sigma_{ref}$ and $\sigma_i$ are the chemical shielding of the reference and atom of interest, respectively. Ring current effects of other subsystems were considered by calculating nuclear independent chemical shifts (NICS) as it was done in Jacob and Visscher (2006).

For comparison of the NMR calculations of the "isoOpt" and "Pa extract" models the *ADF* program with the PBE functional (Perdew et al., 1996b) and triple-ζ-basis set was used.

## 2.4 Calculation of the Environment effect

To calculate the effect of the protein environment on the chemical shifts of the co-factors the following equation was applied,

$$\Delta\delta = \delta\left(\mathrm{CoF}^{34}_{\mathrm{A/B}}\right) - \delta\left(\mathrm{CoF}^{\mathrm{iso}}_{\mathrm{A/B}}\right). \quad (1)$$

In the equation above $\delta\left(\mathrm{CoF}^{34}_{\mathrm{A/B}}\right)$ is the chemical shift of a particular co-factor $\mathrm{CoF}_{\mathrm{A/B}}$ within a protein environment of 3.4 Å, where $\delta\left(\mathrm{CoF}^{\mathrm{iso}}_{\mathrm{A/B}}\right)$ is the chemical shift of a particular isolated cofactor. The difference $\Delta\delta$ is, thus, the effect of the protein environment on the chemical shifts of CoF.

## 2.5 Validation of the NMR Calculations

In this section results are shown that assess the quality of the calculated chemical shifts. Therefore, $^{15}N$ Chemical shifts obtained experimentally by Boxer et al. (1974) and by the authors of this work, which agree very well, are compared to shifts calculated with *ADF* for the "isoOpt" and "Pa extract" model. The data are shown in Fig. S2.3. The calculated chemical shifts are overestimated by about 10 to 20 ppm compared to the experimental value, but correctly predict the trend of the chemical shifts (see also Fig. S2.4). Additionally, the calculated shifts of $N_{II}$ and $N_{III}$ for "Pa extract" are in very good agreement with the experimental values, while $N_I$ and $N_{IV}$ deviate from the experimental results. The reader should note here, that the results for $N_{IV}$ strongly deviate in all four shown lines (see Figs. S2.3 and S2.4).
The deviations in the calculated NMR shifts strongly depend on the geometry of the calculated models. The values calculated for "Pa extract" show good agreement with the experiment especially for nitrogen atoms 2 and 3, while the "isoOpt" values better represent the general trend of the shifts. This shows that a possible error source for the calculated NMR shifts arises from the use of the static crystal structure rather than averaging over conformations accessible during the protein dynamics. This could be assessed by performing

a short molecular dynamics simulation and calculating NMR shifts for an ensemble of structures, which is, however, beyond the scope of this work.

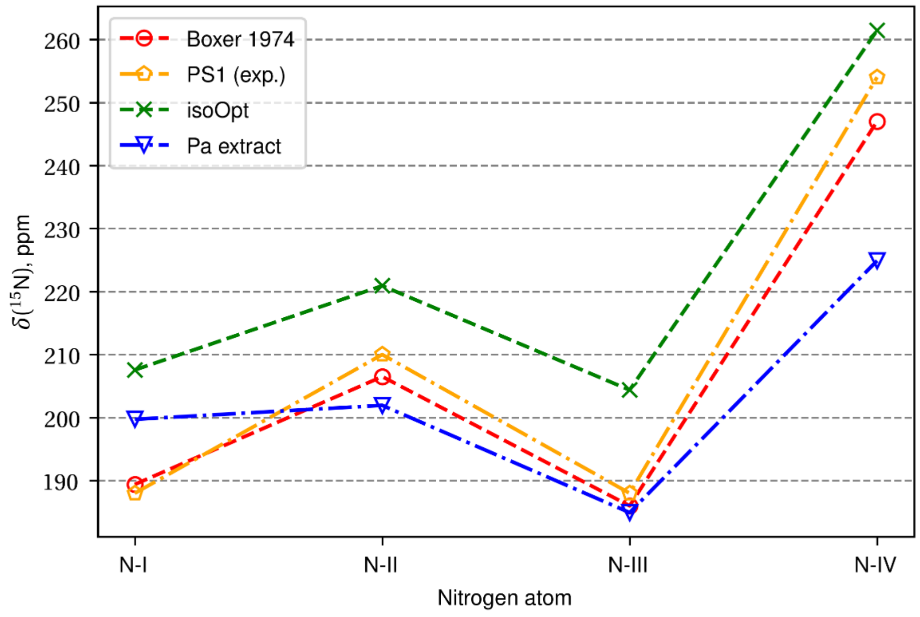


**Figure S2.3: Comparison of $^{15}N$ Chemical shifts obtained experimentally by Boxer et. al. [31] (red) and the authors of this work (orange) with those calculated with ADF for the "isoOpt" (green) and "Pa extract" (blue) model.**

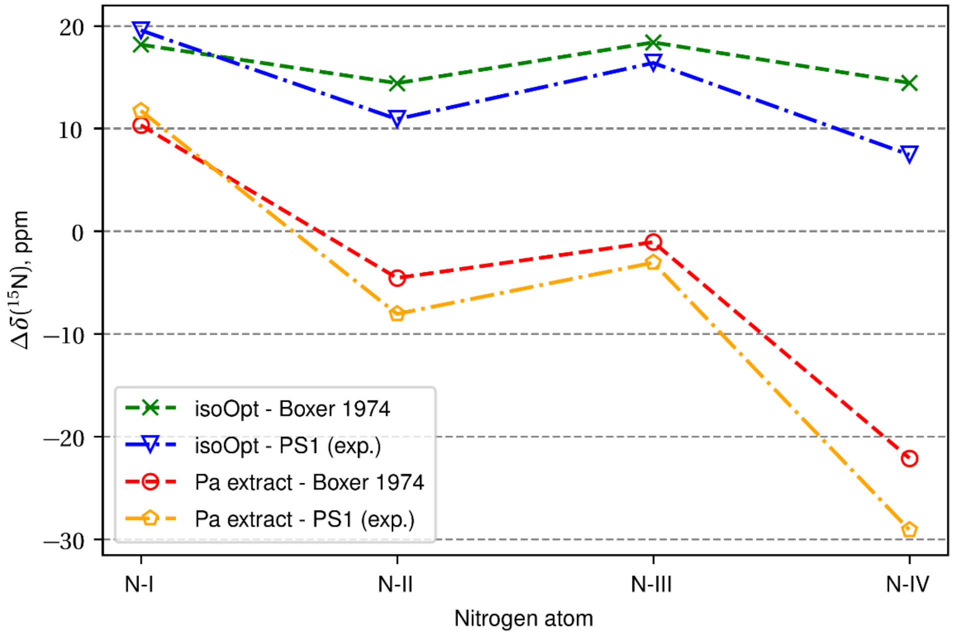


**Figure S2.4: Comparison of differences in the $^{15}N$ Chemical shifts for "isoOpt" and "Pa extract" calculated with ADF with experimental results. The green line shows the difference of the "isoOpt" results and the results of Boxer et. al., while the blue line shows the difference towards the results of this works' authors. The orange and red line show the same differences obtained for the "Pa extract" model.**

## *3. Chemical shifts calculated by quantum-chemical methods*

**Table S2.1: $^{15}$N NMR shifts of $P_A$ and $P_B$ in ppm, including ring current effects induced by the neighboring Chl co-factor. $\Delta\delta$ is the environment effect on the shift in ppm calculated according to Eq. (1). The indices are chosen according to IUPAC (see Fig. S2.2).**

| index | δ($P_A$), ppm | | | δ($P_B$), ppm | | | Δδ, ppm | |
|---|---|---|---|---|---|---|---|---|
| | iso | r32 | r34 | iso | r32 | r34 | $P_A$ | $P_B$ |
| $N_I$ | 184.69 | 191.64 | 191.70 | 181.88 | 186.47 | 186.60 | 7.01 | 4.72 |
| $N_{II}$ | 186.58 | 193.35 | 193.91 | 186.90 | 186.14 | 186.10 | 7.33 | -0.88 |
| $N_{III}$ | 171.88 | 177.28 | 178.38 | 168.13 | 176.57 | 176.52 | 6.50 | 8.39 |
| $N_{IV}$ | 209.79 | 215.12 | 214.86 | 214.71 | 222.92 | 222.67 | 5.07 | 7.96 |

**Table S2.2: $^{15}$N NMR shifts of $A_{0A}$ and $A_{0B}$ in ppm, including ring current effects by the neighboring Chl co-factor. $\Delta\delta$ is the environment effect on the shift in ppm calculated according to Eq. (1). The indices are chosen according to IUPAC (see Fig. S2.2).**

| index | δ($A_{0A}$), ppm | | | δ($A_{0B}$), ppm | | | Δδ, ppm | |
|---|---|---|---|---|---|---|---|---|
| | iso | r32 | r34 | iso | r32 | r34 | $A_{0A}$ | $A_{0B}$ |
| $N_I$ | 176.12 | 181.65 | 183.29 | 181.38 | 185.08 | 185.28 | 7.17 | 3.90 |
| $N_{II}$ | 185.82 | 182.17 | 182.27 | 186.63 | 194.06 | 194.04 | -3.55 | 7.41 |
| $N_{III}$ | 163.71 | 161.67 | 162.00 | 168.43 | 164.43 | 163.34 | -1.71 | -5.09 |
| $N_{IV}$ | 204.25 | 206.59 | 206.43 | 209.16 | 208.23 | 208.81 | 2.18 | -0.35 |

**Table S2.3: $^{13}C$ NMR shifts of $P_A$ and $P_B$ in ppm, including ring current effects induced by the neighboring Chl co-factor. $\Delta\delta$ is the environment effect on the shift in ppm calculated according to Eq. (1). The indices are chosen according to IUPAC (see Fig. S2.2).**

| index | δ($P_A$), ppm | | | δ($P_B$), ppm | | | Δδ, ppm | |
|---|---|---|---|---|---|---|---|---|
| | iso | r32 | r34 | iso | r32 | r34 | $P_A$ | $P_B$ |
| 1 | 138.95 | 140.05 | 140.50 | 142.10 | 142.83 | 142.74 | 1.55 | 0.64 |
| 2 | 118.54 | 120.73 | 119.14 | 121.18 | 123.20 | 122.92 | 0.60 | 1.74 |
| $2^1$ | 11.91 | 12.65 | 11.01 | 12.03 | 12.97 | 12.96 | -0.90 | 0.93 |
| 3 | 132.04 | 132.43 | 134.87 | 135.29 | 136.33 | 136.22 | 2.83 | 0.93 |
| $3^1$ | 134.99 | 137.82 | 138.09 | 135.07 | 138.05 | 138.13 | 3.10 | 3.06 |
| $3^2$ | 108.91 | 113.28 | 118.52 | 112.90 | 116.33 | 116.15 | 9.61 | 3.25 |
| 4 | 146.78 | 149.05 | 149.07 | 146.25 | 146.67 | 146.61 | 2.29 | 0.36 |
| 5 | 126.57 | 130.70 | 131.19 | 125.22 | 123.22 | 123.01 | 4.62 | -2.21 |
| 6 | 150.56 | 154.64 | 154.76 | 151.27 | 154.01 | 153.86 | 4.20 | 2.59 |
| 7 | 125.93 | 128.86 | 128.94 | 128.31 | 131.85 | 131.72 | 3.01 | 3.41 |
| $7^1$ | 13.78 | 14.49 | 14.43 | 14.24 | 16.31 | 16.30 | 0.65 | 2.06 |
| 8 | 139.27 | 140.66 | 141.25 | 138.89 | 140.72 | 140.90 | 1.98 | 2.01 |
| $8^1$ | 27.43 | 27.50 | 27.62 | 27.65 | 27.87 | 27.82 | 0.19 | 0.17 |
| $8^2$ | 29.31 | 30.52 | 31.01 | 23.94 | 24.43 | 24.40 | 1.70 | 0.46 |
| 9 | 146.12 | 147.28 | 147.40 | 147.92 | 149.10 | 149.31 | 1.28 | 1.39 |
| 10 | 122.08 | 122.47 | 122.97 | 118.50 | 118.02 | 119.21 | 0.89 | 0.71 |
| 11 | 145.38 | 146.03 | 146.27 | 149.08 | 151.09 | 151.07 | 0.89 | 1.99 |
| 12 | 128.57 | 130.80 | 132.63 | 128.20 | 136.61 | 137.32 | 4.06 | 9.12 |
| $12^1$ | 16.56 | 14.26 | 14.62 | 16.28 | 16.38 | 16.56 | -1.94 | 0.28 |
| 13 | 158.61 | 158.27 | 159.17 | 152.97 | 155.04 | 155.12 | 0.56 | 2.15 |
| $13^1$ | 248.75 | 249.15 | 247.63 | 254.46 | 255.10 | 254.89 | -1.12 | 0.43 |
| $13^2$ | 65.44 | 66.25 | 66.17 | 80.27 | 79.94 | 79.87 | 0.73 | -0.40 |
| $13^3$ | 181.20 | 179.07 | 181.72 | 180.95 | 183.55 | 183.56 | 0.52 | 2.61 |
| $13^4$ | 55.50 | 58.28 | 57.53 | 56.04 | 57.58 | 57.54 | 2.03 | 1.50 |
| 14 | 134.68 | 135.14 | 135.42 | 134.76 | 135.50 | 135.49 | 0.74 | 0.73 |
| 15 | 115.62 | 115.84 | 114.38 | 115.93 | 115.61 | 115.40 | -1.24 | -0.53 |
| 16 | 152.40 | 151.13 | 150.81 | 154.60 | 152.19 | 151.87 | -1.59 | -2.73 |
| 17 | 52.74 | 52.20 | 53.25 | 58.12 | 58.36 | 58.32 | 0.51 | 0.20 |
| $17^1$ | 40.28 | 39.19 | 39.60 | 40.62 | 40.07 | 39.99 | -0.68 | -0.63 |
| $17^2$ | 27.61 | 27.99 | 27.80 | 40.48 | 40.77 | 40.76 | 0.19 | 0.28 |
| $17^3$ | 180.94 | 183.84 | 184.48 | 180.15 | 182.89 | 183.27 | 3.54 | 3.12 |
| 18 | 52.72 | 52.36 | 52.50 | 56.34 | 56.22 | 56.25 | -0.22 | -0.09 |
| $18^1$ | 35.85 | 34.72 | 35.39 | 33.62 | 33.01 | 33.03 | -0.46 | -0.59 |
| 19 | 151.77 | 150.71 | 151.54 | 149.27 | 146.92 | 146.71 | -0.23 | -2.56 |
| 20 | 97.65 | 95.27 | 97.33 | 99.04 | 99.33 | 99.05 | -0.32 | 0.01 |

**Table S2.4: $^{13}C$ NMR shifts of $A_{0A}$ and $A_{0B}$ in ppm, including ring current effects induced by the neighboring Chl co-factor. $\Delta\delta$ is the environment effect on the shift in ppm calculated according to Eq. (1). The indices are chosen according to IUPAC (see Fig S2.2).**

| index | δ($A_{0A}$), ppm | | | δ($A_{0B}$), ppm | | | Δδ, ppm | |
|---|---|---|---|---|---|---|---|---|
| | iso | r32 | r34 | iso | r32 | r34 | $A_{0A}$ | $A_{0B}$ |
| 1 | 140.07 | 141.91 | 141.12 | 145.16 | 147.14 | 147.40 | 1.05 | 2.24 |
| 2 | 120.74 | 128.20 | 126.83 | 122.73 | 125.23 | 125.20 | 6.09 | 2.47 |
| $2^1$ | 17.79 | 20.40 | 20.91 | 23.28 | 24.39 | 24.38 | 3.12 | 1.10 |
| 3 | 134.76 | 140.04 | 138.55 | 134.53 | 137.11 | 137.36 | 3.79 | 2.83 |
| $3^1$ | 133.33 | 139.08 | 138.38 | 132.17 | 134.58 | 134.34 | 5.05 | 2.17 |
| $3^2$ | 111.83 | 109.27 | 108.08 | 113.10 | 105.55 | 106.44 | -3.75 | -6.66 |
| 4 | 147.63 | 152.40 | 152.39 | 148.45 | 150.42 | 150.58 | 4.76 | 2.13 |
| 5 | 120.67 | 119.79 | 121.92 | 121.99 | 121.76 | 121.84 | 1.25 | -0.15 |
| 6 | 143.59 | 143.36 | 144.32 | 145.21 | 148.05 | 148.00 | 0.73 | 2.79 |
| 7 | 126.31 | 124.20 | 127.69 | 129.45 | 135.17 | 135.01 | 1.38 | 5.56 |
| $7^1$ | 16.31 | 16.14 | 15.73 | 18.65 | 15.88 | 15.87 | -0.58 | -2.78 |
| 8 | 133.86 | 134.20 | 134.30 | 131.45 | 132.34 | 132.13 | 0.44 | 0.68 |
| $8^1$ | 28.25 | 28.47 | 28.59 | 31.18 | 31.00 | 31.06 | 0.34 | -0.12 |
| $8^2$ | 16.06 | 16.47 | 16.54 | 16.67 | 17.81 | 17.82 | 0.48 | 1.15 |
| 9 | 151.98 | 149.11 | 150.02 | 149.24 | 149.91 | 149.75 | -1.98 | 0.51 |
| 10 | 123.45 | 117.26 | 117.92 | 122.52 | 119.43 | 118.69 | -5.53 | -3.83 |
| 11 | 152.65 | 151.87 | 151.94 | 154.83 | 151.17 | 150.85 | -0.71 | -3.98 |
| 12 | 130.53 | 131.48 | 131.47 | 137.49 | 137.19 | 137.40 | 0.94 | -0.09 |
| $12^1$ | 15.39 | 17.20 | 17.17 | 16.29 | 17.60 | 17.61 | 1.78 | 1.32 |
| 13 | 152.61 | 153.14 | 153.10 | 149.77 | 150.34 | 150.30 | 0.49 | 0.53 |
| $13^1$ | 253.12 | 252.05 | 251.55 | 227.16 | 224.87 | 224.39 | -1.57 | -2.77 |
| $13^2$ | 72.68 | 72.06 | 71.87 | 72.95 | 72.68 | 72.44 | -0.81 | -0.51 |
| $13^3$ | 172.22 | 173.48 | 173.31 | 180.10 | 180.02 | 180.44 | 1.09 | 0.34 |
| $13^4$ | 53.62 | 53.81 | 53.73 | 62.72 | 61.70 | 61.94 | 0.11 | -0.78 |
| 14 | 133.67 | 133.47 | 133.43 | 134.57 | 134.07 | 133.97 | -0.24 | -0.60 |
| 15 | 120.96 | 120.25 | 119.98 | 114.90 | 114.32 | 113.20 | -0.98 | -1.70 |
| 16 | 156.54 | 157.17 | 156.43 | 156.09 | 156.03 | 156.25 | -0.11 | 0.16 |
| 17 | 59.18 | 59.51 | 59.20 | 59.78 | 59.74 | 59.82 | 0.02 | 0.04 |
| $17^1$ | 38.69 | 38.65 | 38.73 | 40.36 | 40.43 | 40.36 | 0.04 | 0.00 |
| $17^2$ | 31.78 | 31.84 | 32.17 | 47.74 | 47.93 | 47.85 | 0.39 | 0.11 |
| $17^3$ | 187.37 | 187.83 | 188.20 | 184.85 | 184.07 | 184.11 | 0.83 | -0.74 |
| 18 | 45.98 | 45.83 | 45.89 | 48.66 | 48.01 | 48.03 | -0.09 | -0.63 |
| $18^1$ | 37.81 | 37.97 | 37.80 | 38.05 | 38.25 | 38.24 | -0.01 | 0.19 |
| 19 | 147.37 | 148.34 | 147.45 | 155.57 | 154.99 | 155.27 | 0.08 | -0.30 |
| 20 | 102.19 | 103.22 | 102.57 | 101.67 | 102.96 | 103.16 | 0.38 | 1.49 |

# 4. Effect of the Protein Environment on the calculated NMR shifts

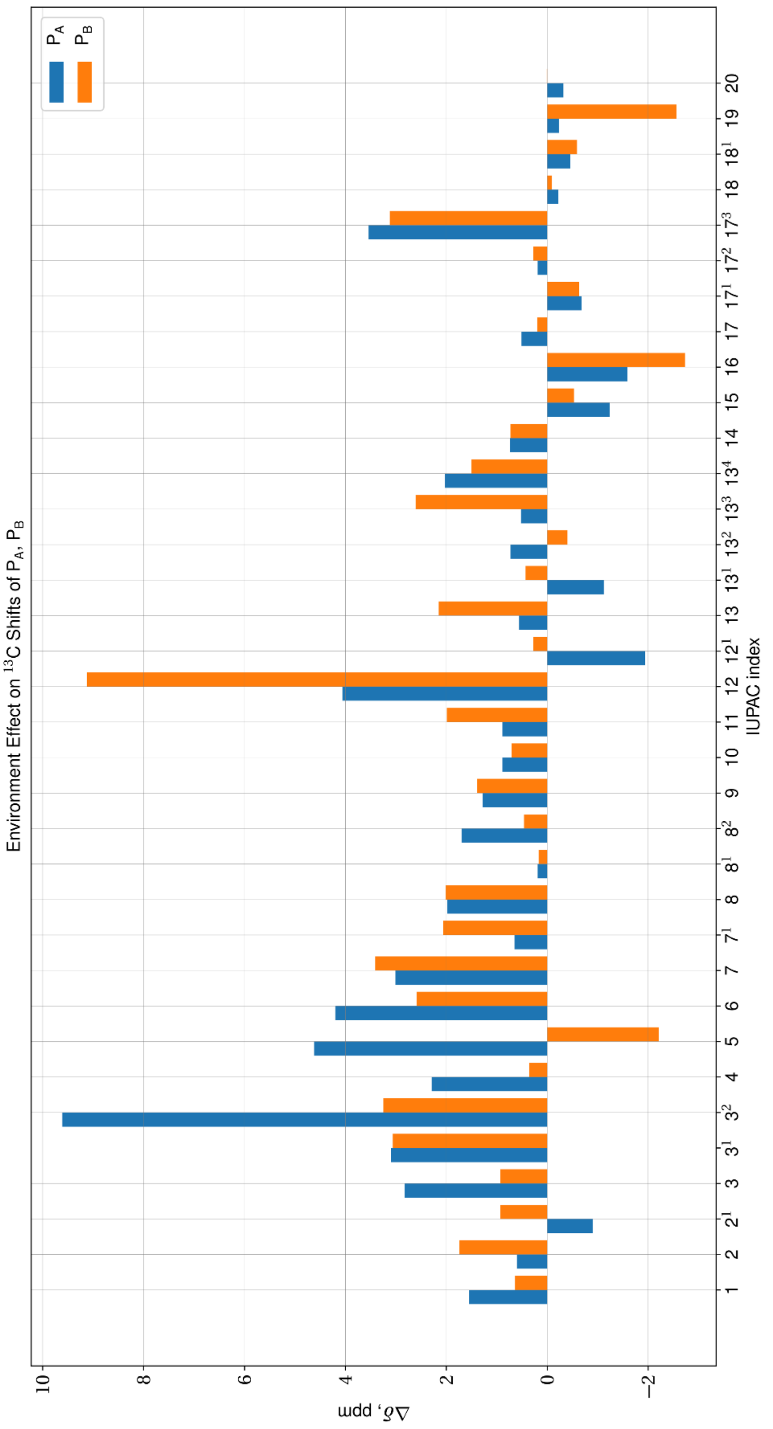


**Figure S3.1: Environment effect on $^{13}$C shifts of $P_A$ and $P_B$.**

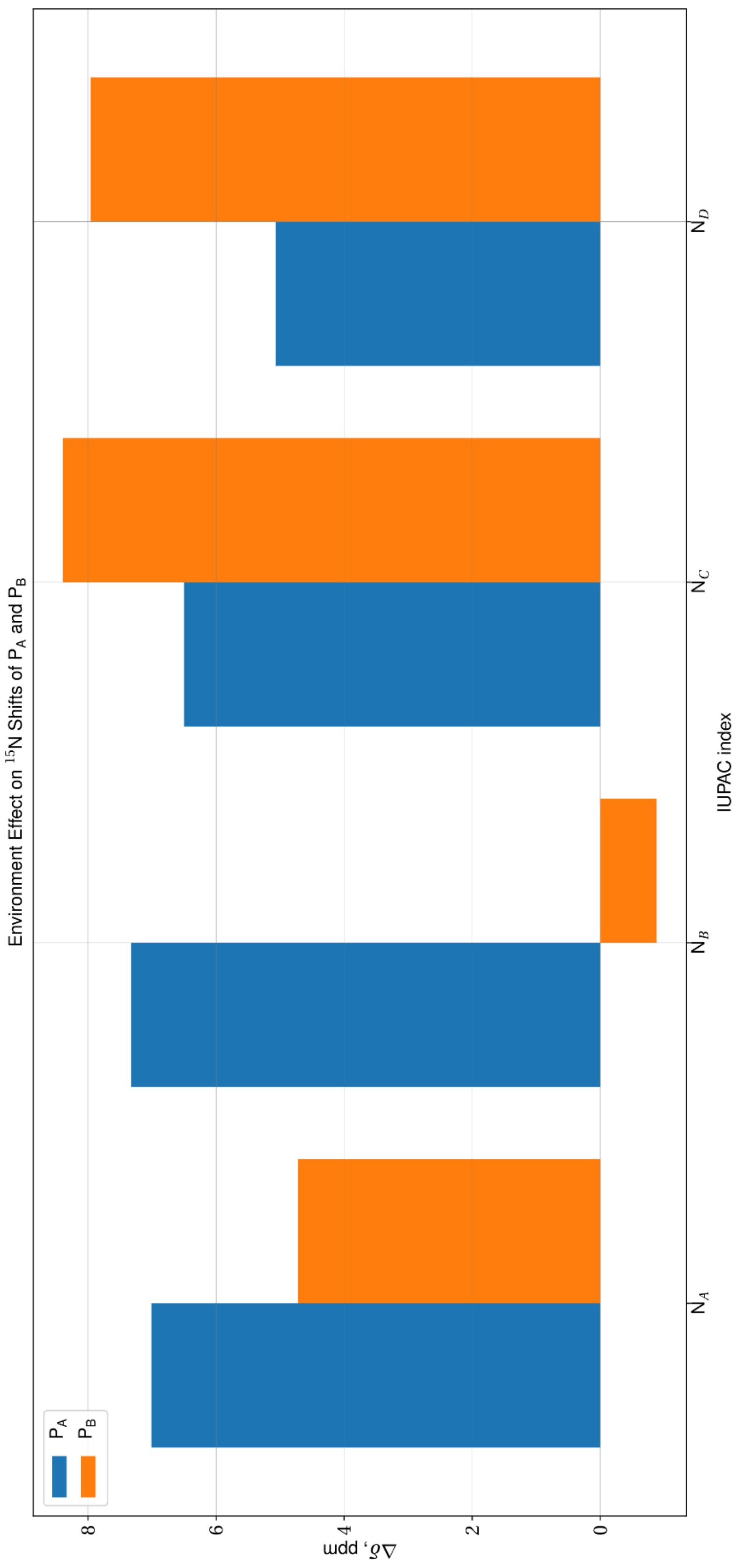


**Figure S3.2: Environment effect on $^{15}N$ shifts of $P_A$ and $P_B$.**

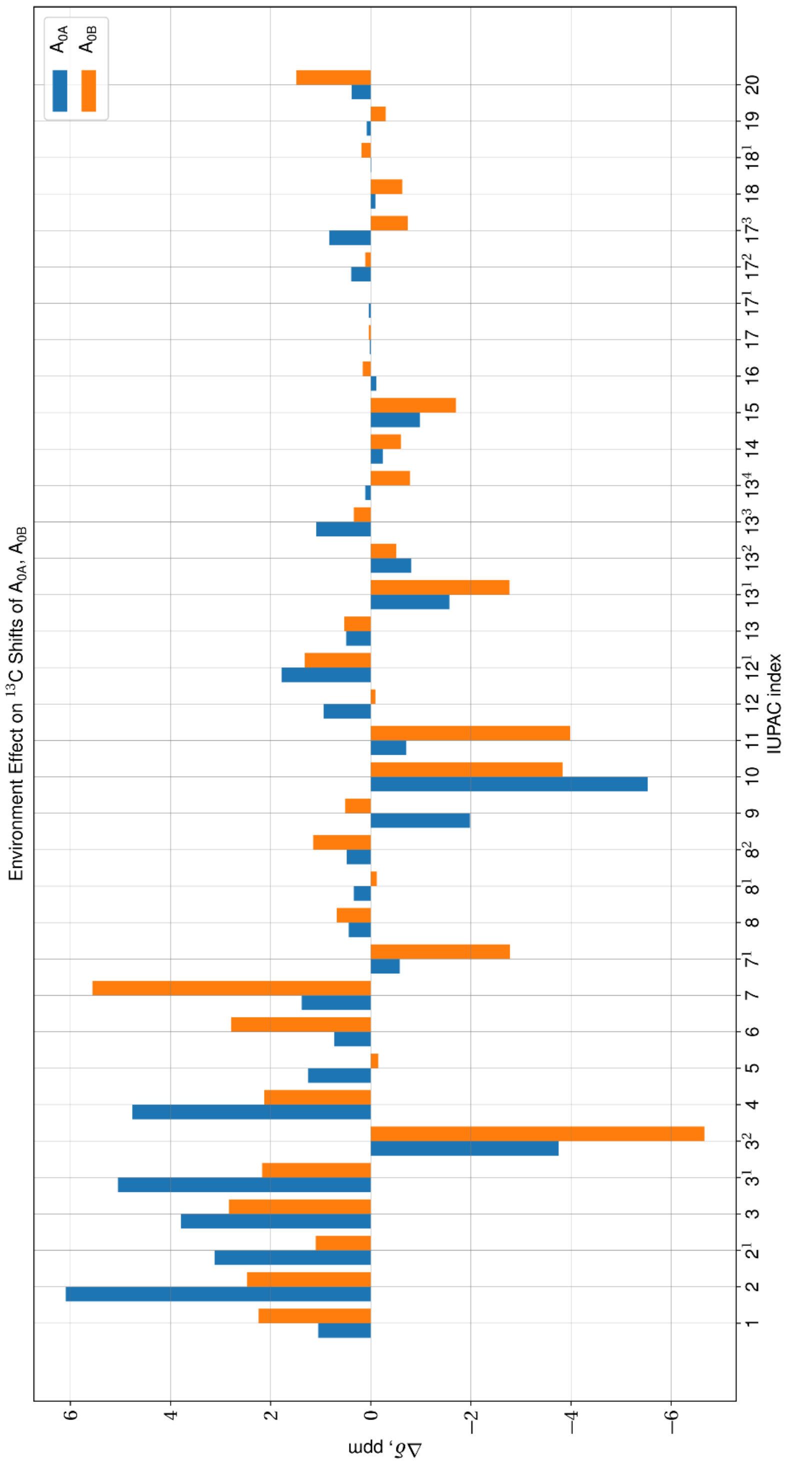


**Figure S3.3: Environment effect on $^{13}C$ shifts of $A_{0A}$ and $A_{0B}$.**

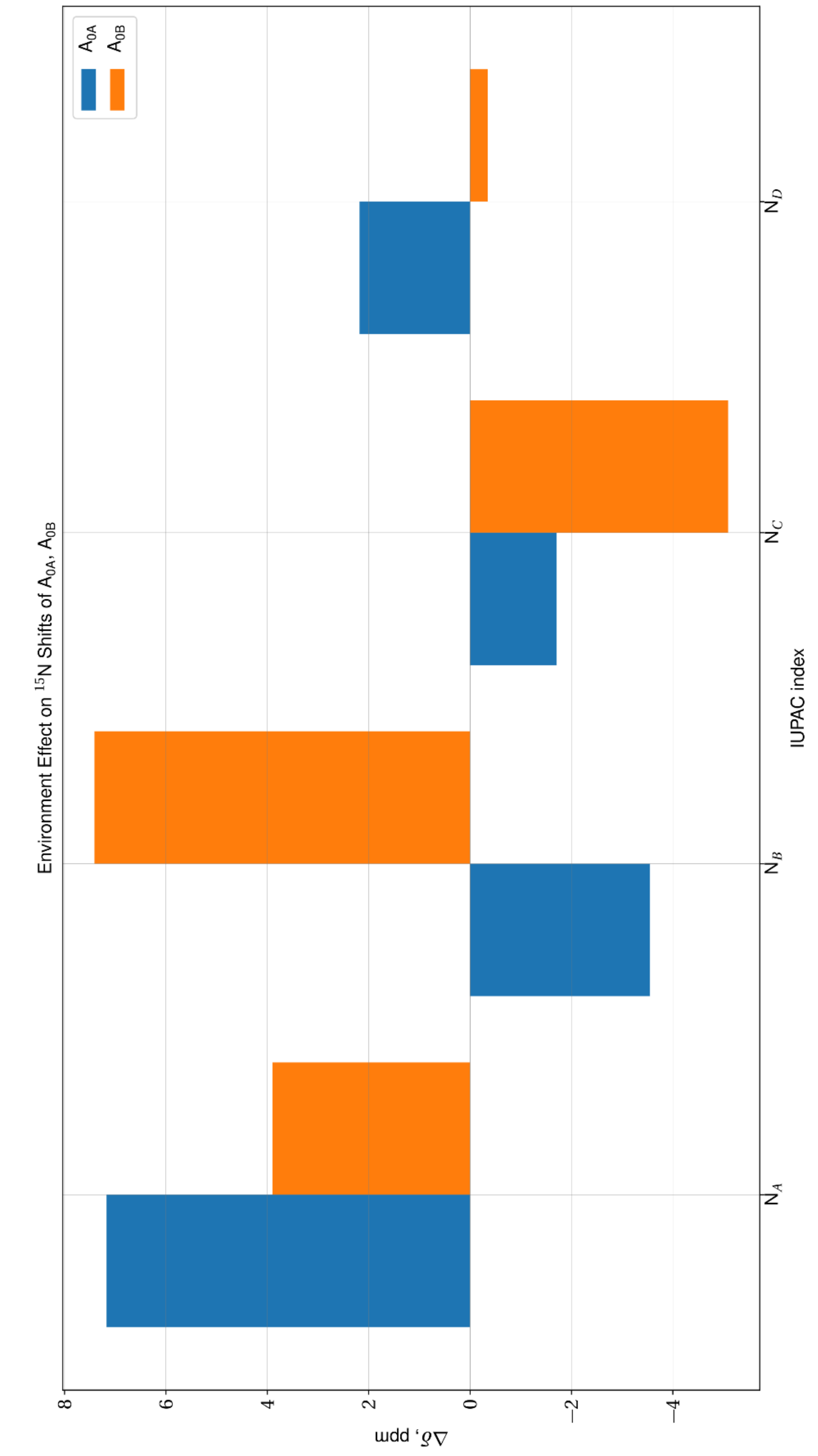


**Figure S3.4: Environment effect on $^{15}N$ shifts of $A_{0A}$ and $A_{0B}$.**

## 5 Graphical Examples of Structures

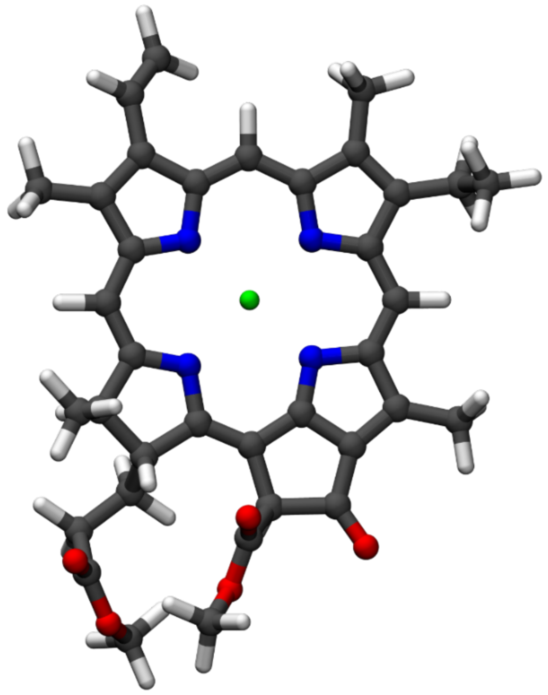

**Figure S4.1: Top view of truncated Chl *a*, "isoOpt".**

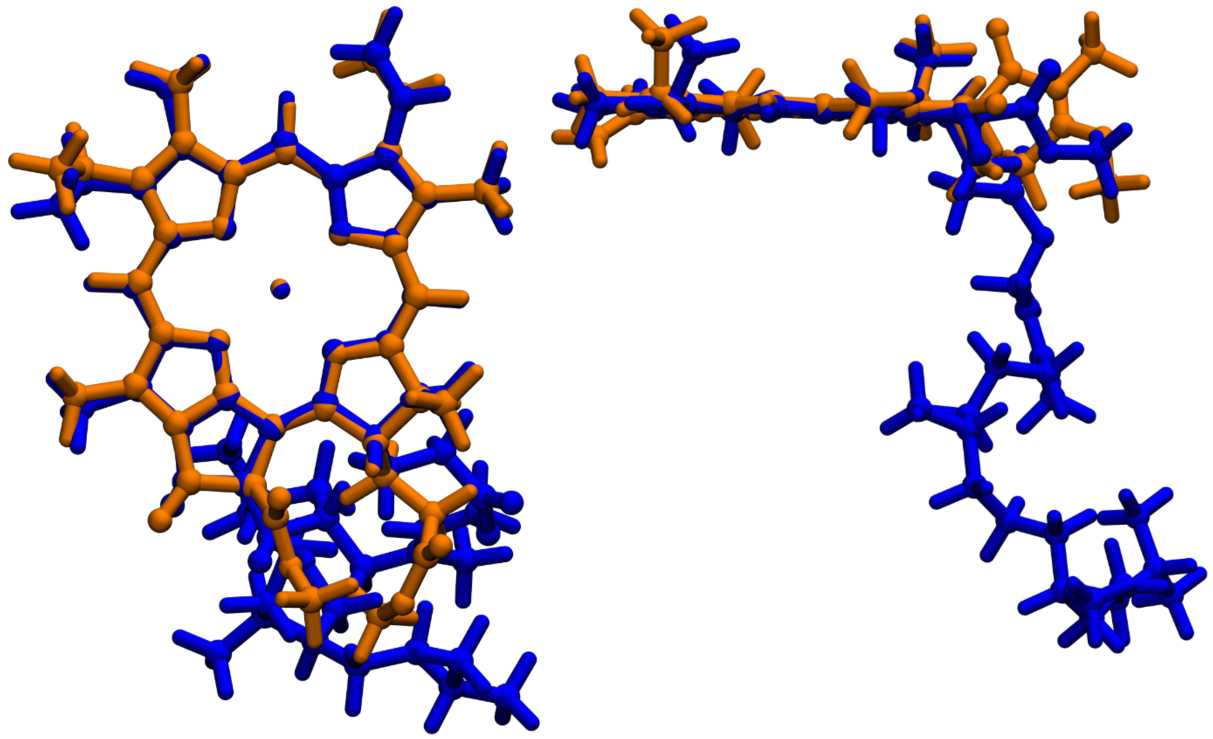

**Figure S4.2: Structural comparison of the fully optimized, truncated Chl *a* "isoOpt" (orange) and "$P_A$ extract" (blue).**

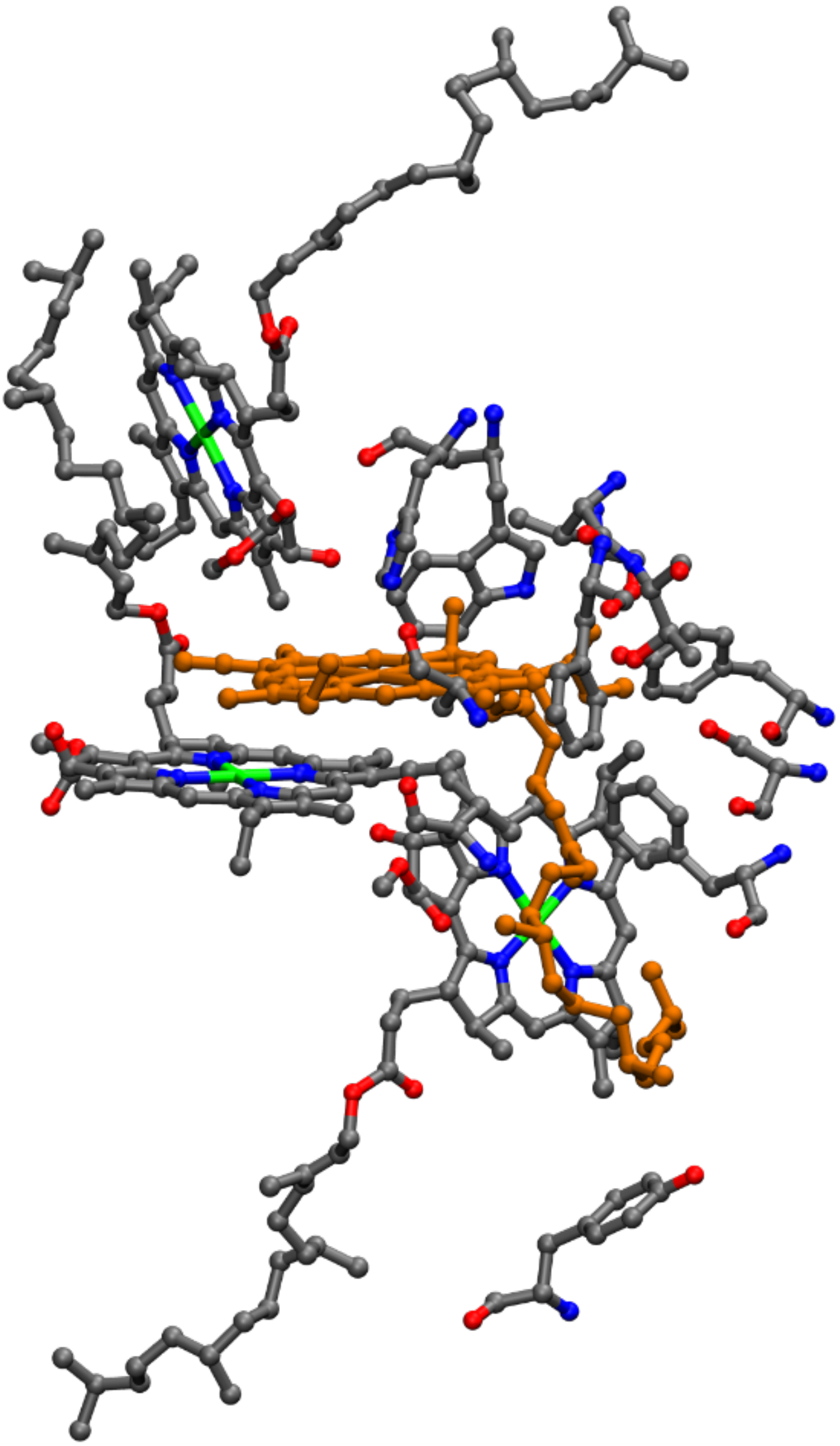

**Figure S4.3: Graphical representation of the model $P_A$ r34. The co-factor $P_A$ is shown in orange, while the protein environment is shown in an element-wise color code. Hydrogen atoms are not shown for the sake of clarity.**

## *(6) Bibliography*


Amsterdam density functional program: http://www.scm.com, last access: 1 September 2020.

Adamo, C. and Barone, V.: Toward reliable density functional methods without adjustable parameters: The PBE0 model, J. Chem. Phys., 100, 6158–6170, doi:10.1063/1.478522, 1994.

Ahlrichs, R., Bär, M., Häser, M., Horn, H., and Kölmel, C.: Electronic structure calculations on workstation computers: The program system turbomole, Chem. Phys. Lett., 162, 165–169, doi:10.1016/0009-2614(89)85118-8, 1989.

Amunts, A., Toporik, H., Borovikova, A., and Nelson, N.: Structure determination and improved model of plant photosystem I, J. Biol. Chem, 285, 3478–3486, doi:10.1074/jbc.M109.072645, 2010.

Artiukhin, D. G., Eschenbach, P., and Neugebauer, J.: Computational investigation of the spin-density asymmetry in photosynthetic reaction center models from first principles, J. Phys. Chem. B, 124, 4873–4888, doi:10.1021/acs.jpcb.0c02827, 2020.

Berman, H. M., Westbrook, J., Feng, Z., Gilliland, G., Bhat, T. N., Weissig, H., Shindyalov, I. N., and Bourne, P. E.: The protein data bank, Nucleic Acids Res., 28, 235–242, doi:10.1093/nar/28.1.235, 2000.

Boxer, S. G., Closs, G. L., and Katz, J. J.: Effect of magnesium coordination on the carbon-13 and nitrogen-15 magnetic resonance spectra of chlorophyll a. Relative energies of nitrogen n.pi.* states as deduced from a complete assignment of chemical shifts, J. Am. Chem. Soc., 96, 7058–7066, doi:10.1021/ja00829a038, 1974.

Gaus, M., Cui, Q., and Elstner, M.: DFTB3: Extension of the self-consistent-charge density-functional tight-binding method (SCC-DFTB), J. Chem. Theory Comput., 7, 931–948, doi:10.1021/ct100684s, 2012.

Gaus, M., Goez, A., and Elstner, M.: Parametrization and benchmark of DFTB3 for organic molecules, J. Chem. Theory Comput., 9, 338–354, doi:10.1021/ct300849w, 2013.

Grimme, S., Antony, J., Ehrlich, S., and Krieg, H.: A consistent and accurate ab initio parametrization of density functional dispersion correction (DFT-D) for the 94 elements H-Pu, J. Chem. Phys., 132, 154104, doi:10.1063/1.3382344, 2010.

Grimme, S., Ehrlich, S., and Goerigk, L.: Effect of the damping function in dispersion corrected density functional theory, J. Comput. Chem., 32, 1456–1465, doi:10.1002/jcc.21759, 2011.

Hanwell, M. D., Curtis, D. E., Lonie, D. C., Vandermeersch, T., Zurek, E., and Hutchison, G. R.: Avogadro: an advanced semantic chemical editor, visualization, and analysis platform, J. Cheminformatics, 4, 17, doi:10.1186/1758-2946-4-17, 2012.

Humphrey, W., Dalke, A., and Schulten, K.: Vmd - Visual Molecular Dynamics, J. Molec. Graphics, 14, 33–38, doi:10.1016/0263-7855(96), 1996.

Jacob, C. R. and Neugebauer, J.: Subsystem density-functional theory, Comput. Mol. Sci., 4, 325–362, doi:10.1002/wcms.1175, 2014.

Jacob, C. R. and Visscher, L.: Calculation of nuclear magnetic resonance shieldings using frozen-density embedding, J. Chem. Phys., 125, 194104, doi:10.1063/1.2370947, 2006.

Keal, T. W. and Tozer, D. J.: The exchange-correlation potential in Kohn–Sham nuclear magnetic resonance shielding calculations, J. Chem. Phys., 119, 3015–3024, doi:10.1063/1.1590634, 2003.

Kollwitz, M. and Gauss, J.: A direct implementation of the GIAO-MBPT(2) method for calculating NMR chemical shifts. Application to the naphthalenium and anthracenium ions., Chem. Phys. Lett., 260, 639–646, doi:10.1016/0009-2614(96)00897-4, 1996.

Kubillus, M., Kubař, T., Gaus, M., Řezáč, J., and Elstner, M.: Parameterization of the DFTB3 method for Br, Ca, Cl, F, I, K, and Na in organic and biological systems, J. Chem. Theory Comput., 11, 332–342, doi:10.1021/ct5009137, 2015.

Lee, H., Lee, C., and Parr, R. G.: Conjoint gradient correction to the Hartree-Fock kinetic- and exchange-energy density functionals, Phys. Rev. A, 44, 768–771, doi:10.1103/PhysRevA.44.768, 1991.

Lembarki, A. and Chermette, C.: Obtaining a gradient-corrected kinetic-energy functional from the Perdew-Wang exchange functional, Phys. Rev. A, 50, 5328–5331, doi:10.1103/PhysRevA.50.5328, 1994.

Mackerell, A. D., Feig, M., and Brooks, C. L.: Extending the treatment of backbone energetics in protein force fields: limitations of gas-phase quantum mechanics in reproducing protein conformational distributions in molecular dynamics simulations, J. Comput. Chem., 25, 1400–1415, doi:10.1002/jcc.20065, 2004.

MacKerell, A. D., Bashford, D., Bellott, M., Dunbrack, R. L., Evanseck, J. D., Field, M. J., Fischer, S., Gao, J., Guo, H., Ha, S., Joseph-McCarthy, D., Kuchnir, L., Kuczera, K., Lau, F. T., Mattos, C., Michnick, S., Ngo, T., Nguyen, D. T., Prodhom, B., Reiher, W. E., Roux, B., Schlenkrich, M., Smith, J. C., Stote, R., Straub, J., Watanabe, M., Wiórkiewicz-Kuczera, J., Yin, D., and Karplus, M.: All-atom empirical potential for molecular modeling and dynamics studies of proteins, J. Phys. Chem. B, 102, 3586–3616, doi:10.1021/jp973084f, 1998.

Perdew, J. P., Burke, K., and Ernzerhof, M.: Generalized gradient approximation made simple, Phys. Rev. Lett., 77, 3865–3868, doi:10.1103/PhysRevLett.77.3865, 1996b.

Perdew, J. P., Chevary, J. A., Vosko, S. H., Jackson, K. A., Pederson, M. R., Singh, D. J., and Fiolhais, C.: Atoms, molecules, solids, and surfaces: Applications of the generalized gradient approximation for exchange and correlation, Phys. Rev. B, 46, 6671–6687, doi:10.1103/PhysRevB.46.6671, 1992.

Perdew, J. P. and Wang, Y.: Electronic structure of solids'91, Academie, Berlin, 1991.

Perdew, J. P., Ernzerhof, M., and Burke, K.: Rationale for mixing exact exchange with density functional approximations, J. Chem. Phys., 105, 9982–9985, doi:10.1063/1.472933, 1996a.

Schäfer, A., Horn, H., and Ahlrichs, R.: Fully optimized contracted Gaussian basis sets for atoms Li to Kr, J. Chem. Phys., 97, 2571–2577, doi:10.1063/1.463096, 1992.

Schäfer, A., Huber, C., and Ahlrichs, R.: Fully optimized contracted Gaussian basis sets of triple zeta valence quality for atoms Li to Kr, J. Chem. Phys., 100, 5829–5835, doi:10.1063/1.467146, 1994.

TURBOMOLE GmbH: TURBOMOLE V7.4.1, 2019.

van Lenthe, E. and Baerends, E. J.: Optimized Slater-type basis sets for the elements 1-118, J. Comput. Chem., 24, 1142–1156, doi:10.1002/jcc.10255, 2003.

Velde, G. T., Bickelhaupt, F. M., Baerends, E. J., Fonseca Guerra, C., van Gisbergen, S. J. A., Snijders, J. G., and Ziegler, T.: Chemistry with ADF, J. Comput. Chem., 22, 931–967, 2001.

Wesolowski, T. A., Shedge, S., and Zhou, X.: Frozen-density embedding strategy for multilevel simulations of electronic structure, Chem. Rev., 115, 5891–5928, doi:10.1021/cr500502v, 2015.

Wesolowski, T. A. and Weber, J.: Kohn-Sham equations with constrained electron density: An iterative evaluation of the ground-state electron density of interacting molecules, Chem. Phys. Lett., 248, 71–76, doi:10.1016/0009-2614(95)01281-8, 1996.

Word, J. M., Lovell, S. C., Richardson, J. S., and Richardson, D. C.: Asparagine and glutamine: using hydrogen atom contacts in the choice of side-chain amide orientation, J. Mol. Biol., 285, 1735–1747, doi:10.1006/jmbi.1998.2401, 1999.
